# Personality From Age 10 to 16 years. A Four-Wave Cohort Study of Development and Sex Differences in the Big Five and Its Facets

**DOI:** 10.1177/01461672251396595

**Published:** 2025-12-26

**Authors:** Silje Steinsbekk, Lars Wichstrøm, Tilmann von Soest

**Affiliations:** 1 Norwegian University of Science and Technology, Trondheim, Norway; 2 University of Oslo, Oslo, Norway

**Keywords:** personality, development, adolescent, children, gender differences

## Abstract

Recent years have witnessed an increased interest in personality development in childhood and adolescence. However, population-based longitudinal studies that examine self-reported personality traits and their facets during these critical developmental periods are scarce. Here, we test the disruption hypothesis, which suggests deviations from standard patterns of personality maturation during adolescence in certain personality domains. Our study extends existing knowledge by examining development and sex differences in self-reported conscientiousness, agreeableness, neuroticism, and their facets from childhood to adolescence. Utilizing the Big Five Inventory, we collected four waves of personality data (ages 10, 12, 14, and 16 years) from a representative birth cohort of Norwegian children (*N* = 805). Our results predominantly support the disruption hypothesis, showing declines in conscientiousness and agreeableness across sexes from age 12, with an increase in neuroticism observed solely for girls. The findings further demonstrate that maturation disruptions vary at the facet level, suggesting a complex developmental process.

## Introduction

Over the past decade, the interest in personality development during childhood and adolescence has grown (Costa et al., 2019). However, compared to adult studies, research examining these formative years remains limited, and there is a particular lack of population-based longitudinal studies to address key questions about personality development in late childhood and adolescence (Costa et al., 2019). While the maturity principle (Caspi et al., 2005) suggests that conscientiousness (i.e., being organized, reliable, responsible) and agreeableness (i.e., being cooperative, friendly) increase with age, and neuroticism (i.e., emotional instability) decreases, Soto et al. (2011) reported evidence for diverging trends during adolescence, supporting a disruption hypothesis, which suggests temporary declines in certain aspects of personality maturation during this period (Denissen et al., 2013; Roberts et al., 2005). They found that conscientiousness and agreeableness dip in early adolescence and rise again later on, along with an increase in girls’ neuroticism (Soto et al., 2011). Subsequent studies have offered mixed results (Branje et al., 2007; Goldstein et al., 2022; Göllner et al., 2017; Klimstra et al., 2009; Luan et al., 2017; Slobodskaya & Kornienko, 2021; Tackman et al., 2017; Tetzner et al., 2023; Van den Akker et al., 2014, 2021). In a recent meta-analysis of longitudinal studies, the authors state that their findings ‘reinforce some but not all aspects of the maturity principle’ (Bleidorn et al., 2022, p. 604). Importantly, studies often begin in adolescence (e.g., Klimstra et al., 2009; Luan et al., 2017), missing potential disruptions in middle childhood. Moreover, most studies of children and adolescents have applied parent or teacher reports (Bleidorn et al., 2022), which only modestly correlate with self-reports (De Fruyt et al., 2006).

While acknowledging the benefits of using other-report, we argue that as youth grow older and gain more autonomy, parents and teachers become less able to observe them in different situations and have less access to their internal states and emotions. Therefore, self-report methods become increasingly important, especially for assessing neuroticism. Another shortcoming of existing studies is the reliance on convenience samples (i.e., non-probability samples), which tend to have poorer generalizability than representative cohort studies such as the present one. This reliance increases the likelihood of yielding biased estimates of population-level development (Jager et al., 2017).

Finally, according to the Five-Factor Model, personality characteristics are hierarchically organized, with facets comprising the lower level and traits representing overarching categories (McCrae & John, 1992). Recent studies have revealed that the maturation of personality from adolescence to adulthood, as well as during adulthood, shows more complex patterns of change at the facet level than at the trait level (Goldstein et al., 2022; Schwaba et al., 2022). To identify the pattern of change from childhood to adolescence, longitudinal studies examining stability and change in personality at the facet levels are needed. Such research can contribute to a more nuanced understanding of personality development (Bleidorn et al., 2022). The present study addresses the above-noted limitations by examining personality development from middle childhood using four waves of self-reported data from a representative birth cohort of Norwegian children. We examine the Big Five Personality traits and their facets and consider potential sex differences. We put the disruption hypothesis to the test, by focusing specifically on the development of conscientiousness, agreeableness, and neuroticism, as the first two traits are expected to drop in adolescence, whereas the latter is expected to increase.

### Personality Maturation and Disruption in Adolescence

The Five-Factor Theory posits that the development of personality traits is driven by an intrinsic impetus toward maturation (Mõttus, 2017). Research shows that, on average, individuals become more conscientious, agreeable, and emotionally stable from late adolescence onwards (Bleidorn et al., 2022), a phenomenon supporting the maturity principle of personality (Caspi et al., 2005). However, it has been theorized that new developmental tasks and demands in adolescents can disrupt personality maturation processes because many adolescents may not yet have developed the capabilities to handle these new demands and increased autonomy. This disruption hypothesis aligns with Social Investment Theory (Roberts et al., 2005), which suggests that personality traits change when individuals have to adapt to new expectations from society. Importantly, because these expectations often differ by gender, patterns of personality development may also diverge. For example, in Western societies, the cultural ideals for females tend to emphasize being caring and agreeable more than those for males (Ellemers, 2018; Koenig & Eagly, 2014). Thus, such gender stereotypes may strengthen the development of agreeableness in girls, reducing the likelihood of disruption.

Adolescence is marked by an actively developing brain, with two key biological processes potentially explaining a dip in maturation during this period. The first is an increase in reward sensitivity, including psychological and neural changes that peak in the late teenage years and are linked to higher gonadal hormone levels during puberty (Shulman et al., 2016). This heightened sensitivity leads to a greater affinity for excitement, novelty, and intense emotions (Shulman et al., 2016). The second process is the maturation of cognitive control, primarily associated with the prefrontal cortex, which develops more slowly than the socio-emotional system (Spear, 2013). Consequently, adolescents are predisposed to high-intensity emotions while their ability to regulate these emotions is still underdeveloped. Given that conscientiousness is intertwined with executive functions and low conscientiousness is related to seeking immediate rewards (Manning et al., 2014), these developmental processes might substantially contribute to the presumed drop in conscientiousness in adolescence. Heightened emotionality and regulation difficulties may also be related to increased levels of neuroticism and drops in agreeableness (Carlo et al., 2012). Notably, girls tend to reach puberty at an earlier age than boys, which may affect both the timing and level of disruption. For example, one might expect girls to exhibit earlier and more pronounced increases in neuroticism relative to boys.

As part of their normative identity development, many adolescents are believed to undergo an identity exploration phase where they question their own values and test new identities, which might result in an identity crisis (Marcia, 1980). Such developments may potentially cause increased levels of neuroticism. Furthermore, the shift in social focus from family to peers is integral to the process of identity formation. This transformation can increase potentially stressful interpersonal events (e.g., Cyranowski et al., 2000), which may further elevate neuroticism and reduce agreeableness. The increasing autonomy and need to set boundaries between oneself and one’s parents that often come with the identity formation process may also cause declines in agreeableness. Finally, emerging sexual and romantic interests and the establishment of intimate relationships may be emotionally challenging and may contribute to a dip in maturation.

In sum, as indicated by the bioecological model (Bronfenbrenner, 1979), personality development is the result of complex reciprocal interactions between an active, evolving child and the elements of his or her environment, which also aligns with the transactional model of development (Sameroff, 2009). Transitioning into adolescence, individuals face an accumulation of developmental challenges, which, as suggested by Social Investment Theory (Roberts et al., 2005), may lead to a temporary drop in development toward personality maturity, and perhaps differently so for boys and girls. On the other hand, adolescence is also a developmental period characterized by psychological and social growth. Here, we investigate which traits, and their corresponding facets, exhibit maturation versus disruption, identifying the ages at which these changes occur and whether they apply to both sexes. Although girls are found to be more conscientious, agreeable, and neurotic compared to boys (Borghuis et al., 2017; De Bolle et al., 2015; de Haan et al., 2017; de Moor et al., 2023; Jones et al., 2022; Klimstra et al., 2009; Slobodskaya & Kornienko, 2021; Soto et al., 2011), the specific timing of these differences remains a matter of debate (De Bolle et al., 2015; Jones et al., 2022). Our study seeks to enhance the current understanding of personality development for several key reasons. First, adolescents displaying high levels of conscientiousness and low levels of neuroticism tend to perform better in school (Poropat, 2009) and face fewer mental health problems than their peers with less adaptive personality traits (Etkin et al., 2022; Strickhouser et al., 2017). Increased knowledge about personality development may make educators and parents better positioned to offer necessary support, ensuring adolescents maintain their academic achievements and adaptive development. Second, to inform and tailor interventions that promote personality traits related to beneficial outcomes (Javaras et al., 2019), we need to identify which changes occur, when, and for whom. Research on personality development in adolescence can identify the vulnerable periods and optimal windows of change and offer insights into those who might benefit most from interventive efforts. As an initial step toward such aims, we test potential disruptions in all Big Five personality traits and their facets during adolescence, and whether they differ between the sexes.

### Existing Empirical Research on Personality Development from Childhood to Adolescence

Given the aim of our study, we focus on research using the Big Five taxonomy that assesses self-reported personality using standardized measures, specifically including studies involving children aged 12 and younger when summarizing relevant findings (Table 1). Self-reports of personality are considered to be valid from age 10 onwards, although measurement errors tend to be lower in older samples (Soto et al., 2008).

**Table 1.** Overview of Relevant Studies.

Authors	Year	Country	Design	Sample	Age at baseline	Measure	Main findings
Borghuis et al.		The Netherlands	Prospective cohort-sequential design. Two cohorts, a randomly selected sample (7 waves), 1 school-sample (6 waves), 1-y follow-ups. *N* = 2,230	The RADAR Young Study (RADAR-Y).	Cohort 1: M_age_=13.5 Cohort 2: M_age_ =16.5	Shortened Dutch version of Goldberg’s Big Five Questionnaire (30 adjectives)	Increasing A, girls showed increasing C, temporal declines in boys’ C, and girls’ emotional stability. C, O, and emotional stability are U-shaped during adolescence.
Branje et al.	2007	The Netherlands	Three annual waves of data from two-parent families with at least two adolescents between the ages of 11 and 15 (*n* = 288).	The Nijmegen Family and Personality Study	The younger adolescents: 12.4 (11.0–14.8 years). The older adolescents: 14.5 (11.4–16.0 years);	Shortened Dutch version of Goldberg’s Big Five Questionnaire (30 adjectives).	Girls increased in O and C, E showed a curvilinear pattern, no changes in emotional stability. No mean changes for boys except for a decrease in E. An increase for girls only.
De Moor et al.	2023	The Netherlands	Prospective cohort-sequential design. Ten waves, age 13-26. Waves 1–6: annual; waves 7–10: biannual. *N* = 497	The RADAR Young Study (RADAR-Y)	M_age_ = 13.01 (*SD* = 0.46)	Shortened Dutch version of Goldberg’s Big Five Questionnaire (30 adjectives).	Linear increase in O and A, whereas E decreased. No changes in C and N.
Etkin et al.	2022	Spain	Three annual waves, *n*=551	School sample	M_age_ = 13.77 (*SD* = 1.29) Age range 12–18.	60-item abridged form of the Short Junior Spanish version of the NEOPI-R	Small decline in N and C, no sign. Changes in E and A, and O not reported.
Göllner et al.	2017	Germany	Four-yearly waves; *n* = 2,761.	The Tradition and Innovation in Educational Systems (TRAIN) study. A randomly selected school sample	M_age_ = 10.7 (*SD* = 0.62)	BFI, self (44 items)-and parent reported (10 items)	C, A, and O decreased; E increased; N was stable
Ibanez et al.	2016	Spn	2 waves, age 12 (2004) and 15 (2007). *N* = 234	School sample	M_age_ = 12.03 (*SD* = 0.60)	The short form of the Junior Spanish NEO-PI-R(JSNEO-S)	A and C decreased, no change for N, E, and O
Jones et al.	2022	USA	Accelerated long design, 7 annual waves (2012–2020). Participants at risk for alcohol use problems were oversampled.	The National Consortium on Alcohol and Neurodevelopment in Adolescence (NCANDA)	Age range: 12–21. M_age_ not reported.	The Ten-Item Personality Inventory (TIPI)	Linear increases in C and A, decrease in O. Boys: linear increase in emotional stability, decrease in E. Girls’ changes were more U-shaped.
Klimstra	2009	The Netherlands	One early to middle adolescent cohort (*n* = 923); one middle to late adolescent cohort (*n* = 390); 5 waves, 1-year intervals.	Randomly selected school sample	Cohort 1: M_age_ = 12.4 years Cohort 1: M_age_ = 16.7 years	Shortened Dutch version of Goldberg’s Big Five Questionnaire (30 adjectives)	A, O, E, and emotional stability increased; little evidence for increases in C.
Luan et al.	2017	Germany	Study 1: 3 waves (ages 12 (1992), 17, 29), *n* = 155; Study 2: accelerated longitudinal design, 3 annual assessments. *n* = 576.	The Munich Longitudinal Study on the Genesis of Individual Competencies (LOGIC)	Study 1: M_age_ = 12 Study 2: Divided into five cohorts, M_age_ ranging from 11.5 (*n* = 99) to 15.5 (*n* = 102)	Study 1: Bipolar adjective pairs capturing Big 5, 8 items each trait. Study 2: Shortened Dutch version of Goldberg’s Big Five Questionnaire (30 adjectives).	N and E were stable, A, C, and O increased (Age 12–29). Personality maturation from childhood to young adulthood with disruptions during adolescence.
Pullman et al.		Estonia	2 waves (2001 and 2003), *n* = 876.	Selected school sample	M_age_ = 14.9 (*SD* = 2.0) Age range: 12–18 (reported in Allik et al., 2004).	NEO-FFI	Age 12–14: N and A decreased, E increased, O and C were stable; age 14–16: N decreased, E and O increased, and A and C were stable; 16–18: O increased, no further changes
Schultz et al.		USA	Accelerated longitudinal design, five cohorts, 4 yearly waves, grades 7th–10th, *n* = 951	The Oregon Youth Substance Use Project, school sample	M_age_ _*=*_ 13.12 (*SD* = .37)	Students rated themselves (compared to others) on two adjectives from the BFI dimensions.	C increased (primarily from grade 9 to 10), N decreased (grade 8–9), and O decreased, E and A were stable
Slobodskaya & Kornienko	2021	Russia	Cross-sectional design. *n* = 4,663. Divided into five age groups (1st: 10–11 y)	Convenience sample	M_age_ = 14.60 (*SD* = 1.55) Age range:10–19	Inventory of Child Individual Differences-Short Version (ICID-S)	Increase in self-reported N, E, O, and disagreeableness, in adolescence, no change in C.
Soto et al.		USA	Cross-sectional design. *N* = 1,267,218, with at least 945 participants at each year of age.	Convenience sample, online survey	M_age_ = 25.7 (*SD* = 10.9) Age range: 10–65	BFI	C and A dip in early adolescence, before rising again; E and O declined; an increase in female N, a decrease in boys’ N
Tackman et al.	2017	USA	3 × 3 years of follow-ups, ages 10 (*n* = 90), 13 (*n* = 56), 16 (*n* = 44).	Convenience sample (using flyers, etc.)	Age 10 (exact mean age not reported)	8-item EPSI-C (Conscient. only)	C decreased from age 10 to 13, before increasing from age 13 to 16.
Tetzner et al.	2023	Germany	3 waves: T1: spring 2011; T2: autumn 2011; T3: spring 2014. *N* = 1,662.	The multi-cohort longitudinal Berlin study, a randomly selected school sample	M_age_ = 11.68 (*SD* = 0.63)	The short scale of the Big Five Inventory (BFIS) (15 items)	Increase in E and A, no short term, but only long-term increases in C and N. O: short-term decrease, before increasing. Girls increased more in N than boys.
Van den Akker et al.	2014	Belgium	5 waves, T1 (2001), T2 (2004), T3 (2007), T4 (2009), T5 (2012). *N* = 510 (self-report from T2 onwards)	The Flemish Study in Parenting, Personality and Development. Stratified sample, randomly selected school sample.	M_age_ T1: 7.5 years. Self-report included from T2 onwards, but M_age_ for T2 is not reported.	The Hierarchical Personality Inventory for Children (HiPIC), a 144-item self-report (originally a parent-reported questionnaire)	C decreased from age 10 to 15, then increased. Girls evinced a higher decrease in emotional stability than boys up to age 17. Imagination decreased from age 9 to 15, then increased. E decreased from age 9. Benevolence decreased (9–14), then increased.
Van den Akker et al.	2021	USA	Cross-sectional design. *N* = 2,640	The Texas Twin Project (families with twins)	M_age_ = 13.6 (range:6.94–21.29; 94%: 8–18 years)	BFI, self-report	A and C declined from age 10 to 14 in girls, A declined one year later for boys, and C declined from age 8. A and C increased from age 14 to 16. N increased in girls, decreased in boys. E decreased from age 11in boys, and increased from age 16 in girls. Decrease in O from age 12.
Van Dijk et al.	2020	The Netherlands	Two cohorts, 6 waves over 9 years (12–25) (T1–T5 yearly waves; T6 + 5 years).	Randomly selected schools	Cohort 1: M_age_ = 12.7 Cohort 2: M_age_ = 16.9	Shortened Dutch version of the Quick Big Five questionnaire (30 items)	Mean levels mainly increased from age 12 to 25, but slopes were non-significant for emotional stability and E in the younger cohort. C was stable from early to middle adolescence.

*Note.* Only studies using the Big Five taxonomy and assessing self-reported personality using standardized measures and including children aged 12 and younger are included (Borghuis et al., 2017; Branje et al., 2007; de Moor et al., 2023; Etkin et al., 2022; Göllner et al., 2017; Ibáñez et al., 2016; Jones et al., 2022; Klimstra et al., 2009; Luan et al., 2017; Pullmann et al., 2006; Slobodskaya & Kornienko, 2021; Soto et al., 2011; Tackman et al., 2017; Tetzner et al., 2023; Van den Akker et al., 2014, 2021; Van Dijk et al., 2020).

As shown in Table 1, several studies show conscientiousness to decline in early adolescence, followed by a subsequent increase in later adolescence, thus supporting the disruption hypothesis. This was first reported in Soto et al.’s (2011) large-scale cross-sectional study and has later been replicated in a German sample covering ages 11–14 (Göllner et al., 2017), in a Belgian sample (Van den Akker et al., 2014), in two US studies (Tackman et al., 2017; Van den Akker et al., 2021), in two Spanish studies (Etkin et al., 2022; Ibáñez et al., 2016), and in a Dutch study focusing on boys (Klimstra et al., 2009). In the German sample (Göllner et al., 2017), girls were found to be more conscientious than boys from fifth grade already, whereas Soto et al. (2011) did not find sex differences in conscientiousness to be evident until emerging adulthood. Similar to conscientiousness, several studies have reported that agreeableness evinces a dip in early adolescence before increasing again (Göllner et al., 2017; Ibáñez et al., 2016; Pullmann et al., 2006; Slobodskaya & Kornienko, 2021; Soto et al., 2011; Van den Akker et al., 2014, 2021). Soto et al. (2011) found that boys displayed lower scores than girls from age 10 onwards. More recent research capturing the transition to adolescence also reported girls to be more agreeable than boys and that this sex difference did not change with age (Göllner et al., 2017; Slobodskaya & Kornienko, 2021; Tetzner et al., 2023; Van den Akker et al., 2014). As further displayed in Table 1, some studies show neuroticism to increase in girls in the transition to adolescence, with slight decreases in boys (Jones et al., 2022; Soto et al., 2011; Van den Akker et al., 2014, 2021). However, one study found that neuroticism also increased in boys, but less so than in girls (Tetzner et al., 2023).

It should be acknowledged, though, that some research also supports the maturation hypothesis. For example, Tetzner et al. (2023) found a long-term increase in conscientiousness among individuals aged 12 to 15, as did Schultz et al. (2024), leaning on four waves of data capturing grade 7 to 10. Similarly, Jones et al. (2022) reported a linear increase, as did Luan et al. (2017) in their study of individuals aged 12 to 29. However, other studies found conscientiousness to increase in girls only (Borghuis et al., 2017; Branje et al., 2007), and several others have reported conscientiousness to remain stable during adolescence (de Moor et al., 2023; Pullmann et al., 2006; Slobodskaya & Kornienko, 2021; Van Dijk et al., 2020). Some findings regarding agreeableness are also in accordance with the maturation hypothesis, showing an increase in agreeableness during adolescence (Borghuis et al., 2017; de Moor et al., 2023; Jones et al., 2022; Klimstra et al., 2009; Luan et al., 2017; Tetzner et al., 2023; Van Dijk et al., 2020), with some reporting this increase to be evident in girls only (Branje et al., 2007). Yet, other studies have found that agreeableness remains stable during adolescence (Etkin et al., 2022; Schultz et al., 2024). The maturation hypothesis is also partly confirmed when it comes to neuroticism, with some reporting a slight decline in neuroticism (Etkin et al., 2022; Klimstra et al., 2009; Pullmann et al., 2006; Schultz et al., 2024), whereas a study of German youths assessed annually from ages 11 to 14 observed no change in neuroticism (Göllner et al., 2017), a finding echoed by several other studies (Branje et al., 2007; de Moor et al., 2023; Ibáñez et al., 2016; Luan et al., 2017).

Importantly, as detailed in Table 1, the age range and the number of follow-ups included in these studies vary substantially, making comparisons across studies challenging. Many studies are not well-positioned to identify when potential disruptions appear due to long time intervals between assessments, few follow-ups, or because they did not include participants younger than 12 years. As noted above, to inform and tailor interventions that promote personality traits related to beneficial outcomes (Javaras et al., 2019), we need to identify which changes occur, when, and for whom.

A recent meta-analytical study of personality development throughout the lifespan reported that the mean level of most traits modestly increases from age 10 to 20. The authors noted that only conscientiousness declined in early adolescence before increasing (Bleidorn et al., 2022), with no other disruptions in maturity observed. Importantly, though, because results were collated across ages 10 to 20, the findings cannot precisely pinpoint the timing of this dip. The authors also emphasize the need for longitudinal studies that capture development and change at the facet level of traits to refine our understanding of personality development (Bleidorn et al., 2022). Furthermore, despite the substantial biological, psychological, and social changes occurring during the transition from childhood to adolescence, fewer than a handful of prospective studies have captured this period, and all of them have relied on parent or teacher reports. Using a cohort-sequential design (i.e., data from different cohorts are combined to approximate developmental trajectories), de Haan et al. (2017) reported linear changes in several of the parent-reported conscientiousness facets, thus contradicting the disruption hypotheses. However, their findings on the facets of emotional stability (i.e., neuroticism) supported the hypothesis, at least for boys, as they observed U-shaped changes in emotional stability.

Examining ages 9 to 13, Brandes et al. (2021) also found that the mother-reported facets within each domain evinced different patterns of change in terms of extent and direction. For example, the ‘fear’ facet of neuroticism decreased, while ‘shyness’ evinced no change. In contrast to the findings of de Haan et al. (2017) though, they did not find the facets of neuroticism to differ between the genders, whereas Prinzie and Dekovic (2008) found that girls (ages 6–9 at baseline) showed a larger decline in the negative emotionality facet of self-confidence than boys over the 3-year study period, reported by teachers. The latter study also revealed that the conscientiousness facet ‘achievement motivation’ decreased, whereas the other conscientiousness facets did not, with no differences between the genders (Prinzie & Dekovic, 2008). The two neuroticism facets also evinced different developments as ‘self-confidence’ decreased, and the ‘anxiety’ facet remained stable. A large cross-sectional study of self-reported personality also reported different developmental trends for the within-domain facets of conscientiousness and neuroticism (Soto et al., 2011).

In summary, despite the inconsistent patterns of change, studies on other-reported personality suggest that facets within each domain exhibit distinct developmental trajectories, indicating that personality development is even more complex at the facet level than at the trait level. Taking sex differences into account adds further complexity to this development. Also, by increasing age and autonomy, parents become less positioned to correctly observe and report (i.e., other report) on their offspring’s internal states (e.g., neuroticism); thus, self-report methods of facets may be more valid. Because previous studies have relied on other-reported facets, we therefore extend existing research by examining facet-level development using self-reported data, while also testing for potential sex differences.

### The Present Study

In sum, while acknowledging the contributions of existing research on personality development, we highlight the fact that many studies rarely include pre-adolescents and that there is a need for longitudinal studies examining changes at the facet level using self-reports (Bleidorn et al., 2022). This gap in the literature motivates the present study. Our goal is to rigorously test the disruption hypothesis. To achieve this, we analyze self-reported personality traits and facets across four waves of data, spanning middle childhood to middle adolescence, addressing how self-reported Big Five personality traits and their facets develop from age 10 to 16 years. We also assess potential sex differences, as accounting for sex is essential to providing a more comprehensive understanding of developmental patterns. Given that both the maturation and the disruption hypotheses have received empirical support, we test the following contrasting hypotheses: (a) conscientiousness, agreeableness, and neuroticism decline during adolescence (i.e., the disruption hypothesis) and (b) conscientiousness, agreeableness, and neuroticism increase steadily throughout adolescence.

## Method

### Transparency and Openness

Data are not available due to restrictions related to participant consent and because the study is still ongoing. The structural equation modeling program Mplus 8.5 was used for all analyses (Muthén & Muthén, 1998–2017), and scripts and outputs are available at OSF. This study’s design and analysis were preregistered in AsPredicted^1^, and deviations from the preregistration are displayed in the Online Supplementary Material (Table S1).

### Procedure and Participants

The present study uses data from the ongoing Trondheim Early Secure Study (TESS), which is approved by the Regional Committee for Medical and Health Research Ethics, Central Norway (2019/509).

In 2007 and 2008, all 4-year-olds born in Trondheim, Norway (*N* = 3,456) and their parents were invited to participate in the TESS project, which aims to examine psychosocial development, mental health, and health behavior from early childhood onwards (Steinsbekk & Wichstrøm, 2018). Invitations were sent by mail, together with the Strengths and Difficulties Questionnaire (SDQ), version 4-16 (Goodman et al., 2000), a screening assessment for emotional and behavioral problems, which the parents brought when they attended the mandatory health check at age 4 at the community healthcare centers.

Nearly all children (97.2%) attended the health check. In accordance with the procedures approved by the Regional Committee for Medical and Health Research Ethics, Central Norway, parents were informed about the study by the healthcare nurse (*n* = 3,016). Of those who were eligible to participate, the healthcare nurses missed asking 5.2%. Parents with insufficient proficiency in Norwegian to fill out the SDQ screening were excluded (*n* = 176). In all, 82.1% (*n* = 2,475) provided written consent to participate. Due to TESS’s overall aim to examine mental health, children with higher SDQ scores were oversampled to increase statistical power, which is accounted for in the analyses (i.e., weighting with inverse probabilities). More specifically, children were allocated to four strata according to their SDQ scores (cutoffs: 0–4, 5–8, 9–11, and 12–40), and the probability of selection increased with increasing SDQ scores (0.37, 0.48, 0.70, and 0.89 in the four strata, respectively). Based on this procedure, we selected 1,250 participants, and 1,007 (79.8%) of them attended the university clinic for the first assessment (age 4 years). The sample is representative of the Norwegian population in terms of parents’ level of education (Statistics Norway, 2023) and family situation (i.e., parents married, one-parent household, etc.) (Statistics Norway, 2017). At baseline (age 4), 88.9% of the biological parents were married or cohabiting, 92.8% of the parents had completed senior high school or higher education, and the majority were ethnic Norwegians (mothers: 90.4%; fathers: 88.2%).

Since age 4, participants have been reassessed at the university clinic on a biennial basis. In accordance with Norwegian law, participating children were informed about the study at age 12 and gave written consent themselves at age 16. Personality was assessed from age 10 onwards. The present study thus captures four assessment points: age 10 (2013/2014, *n* = 704, 52.1% girls, 47.9% boys, *M*_age_ = 10.51, *SD* = 0.17), age 12 (2015/2016, *n* = 666, 51.7% girls, 48.3% boys, *M*_age_ = 12.49, *SD* = 0.15), age 14 (2017/2018, *n* = 635, 52.9% girls, 47.1% boys, M_age_ = 14.35, *SD* = 0.16), and age 16 (2019/2020, *n* = 666, 55.0% girls, 44.9% boys, *M*_age_ = 16.98, *SD* = 0.31). The analytical sample constitutes participants with valid personality data on at least one assessment point (*N* = 805).

### The Big Five Personality Inventory

Personality traits were assessed by the Norwegian version of the self-reported Big Five Personality Inventory (BFI), which consists of 44 items assessing Conscientiousness (9 items), Agreeableness (9 items), Neuroticism (8 items), Extraversion (8 items), and Openness (10 items) (Soto et al., 2008). Response options range from 1 (*disagree*) to 5 (*agree*). We also used the 10 specific facet traits described by Soto and John (2009), namely Order (2 items) and Self-discipline (5 items), subsuming Conscientiousness; Altruism (4 items) and Compliance (3 items) subsuming Agreeableness; Depression (2 items) and Anxiety (4 items) subsuming Neuroticism; Assertiveness (5 items) and Activity (2 items) subsuming Extraversion; Aesthetics (3 items) and Ideas (5 items), subsuming Openness. The internal consistency of all traits and facets is displayed in Table 2.

**Table 2.** Internal Consistency of the BFI Traits and Their Facets.

		Internal Consistency (Cronbach’s α)			
BFI Traits and Facets	No. of items	Age 10	Age 12	Age 14	Age 16
Conscientiousness	9	.65	.72	.77	.76
Order	2	.36	.38	.46	.49
Self-discipline	5	.49	.53	.60	.61
Agreeableness	9	.64	.71	.72	.71
Altruism	4	.46	.55	.59	.61
Compliance	3	.36	.41	.41	.42
Neurotcism	8	.59	.72	.81	.83
Anxiety	4	.50	.69	.75	.77
Depression	2	.31	.38	.49	.60
Extraversion	8	.54	.67	.75	.81
Assertiveness	5	.42	.57	.64	.70
Activity	2	.52	.53	.60	.61
Openness		.70	.74	.74	.76
Aesthetics	3	.67	.74	.77	.71
Ideas	5	.46	.53	.58	.60

We examined measurement invariance across age using a series of confirmatory factor analyses (see Tables S2 and S3 in the Online Supplemental Material). The results indicated configural and metric invariance for all five personality traits. However, model fit indices suggested less than optimal full scalar invariance for agreeableness, extraversion, and openness, but we achieved partial scalar invariance when freeing up three intercepts for each of the three personality traits. Similarly, analyses of the personality facets showed satisfactory metric invariance, but substantially reduced model fit for several facets at the scalar level, indicating challenges in establishing full scalar invariance (see Online Supplement). We also assessed measurement invariance across sex (see Tables S4 and S5 in the Online Supplement), and these analyses largely supported configural, metric, and scalar invariance across sex for all traits and facets.

### Sex

Sex was coded based on the participant’s national ID number, which is based on sex assigned at birth.

### Statistical Analyses

Sex differences in the mean level of personality traits were tested by regressing each personality trait on sex at ages 10, 12, 14, and 16. To provide information about the overall development of personality traits from age 10 to 16, latent growth curves were estimated for each trait separately. We estimated linear and curvilinear growth curves where an additional quadratic slope was included. Moreover, sex was included as a predictor of the intercept and growth factors to estimate sex-specific personality trajectories. By regressing the growth parameters on sex, we were able to specify model-implied, sex-specific growth estimates, allowing us to model sex-specific growth curves. These estimates were derived by altering the coding of the sex variable (e.g., coding boys as 0 and girls as 1 and vice versa).

To provide more detailed information about personality changes across age, we constructed latent change score models for each personality trait, modeling changes from one point to the next within a single overall model. To examine sex-specific changes, we additionally regressed latent change scores on sex (see Figure 1 for a graphical display of the model). This approach allowed us to estimate the mean change in personality traits from age 10 to 12, from age 12 to 14, and from age 14 to 16. Similar to our growth curve framework, by regressing the latent change scores on sex, we specified model-implied, sex-specific change estimates. We used the same analytical approach when examining personality facets. We performed post-hoc power analyses by means of Monte Carlo simulations with the *RAMpath* package version 0.5.1 (Zhang & Liu, 2018) in R. The results indicated that a small-sized latent mean change in personality traits (Cohen’s *d* = 0.1) between time points could be detected with an 80% power, given the available sample size and a significance level of *p* < .05.

**Figure 1.**
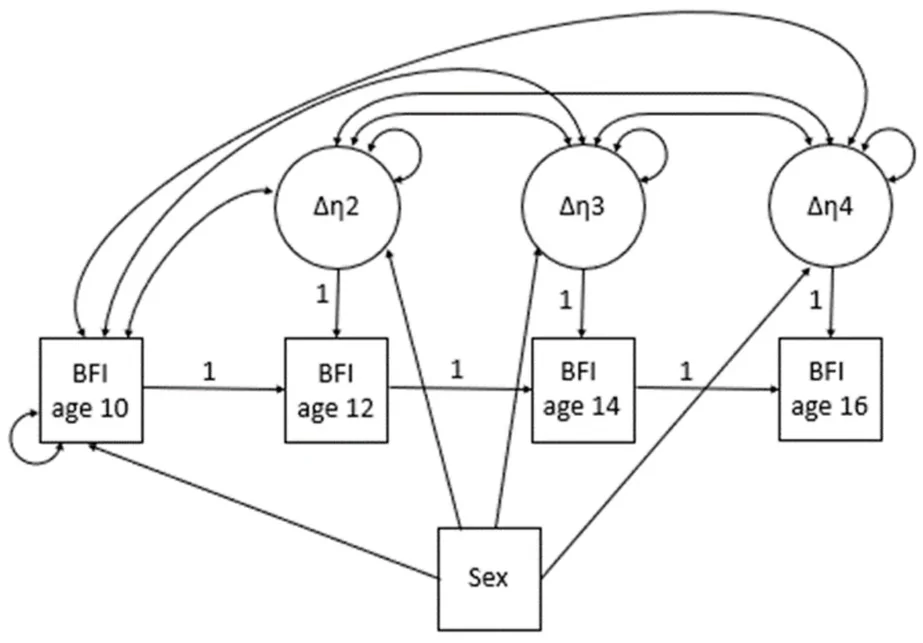
Latent change score model estimating change in personality traits with sex as a predictor of change. Mean structure not depicted. *Note.* BFI = Big Five Inventory.

Model fit was evaluated based on the following indexes/tests and recommendations: The Confirmatory Fit Index (CFI) (>0.90); The Tucker-Lewis Index (TLI) (>0.90); The Root Mean Square Error of Approximation (RMSEA) (<0.06) (Hu & Bentler, 1999); and for comparison between nested models we used the Satorra-Bentler scaled chi-square test (Satorra & Bentler, 2001).

We report all manipulations, measures, and exclusions conducted in the present study.

All analyses were weighted using a factor corresponding to the number of persons in the stratum in the population divided by the number of participants in that stratum to account for cluster sampling, applying a sandwich estimator. We applied full information maximum likelihood (FIML), which is recommended in the methodological literature as one of the most appropriate methods of handling missing data, with similar performance to multiple imputation procedures (Enders, 2022). FIML is used under the assumption that data are missing at random (MAR).

Multivariate logistic regression analyses showed that the study variables did not predict attrition, with two exceptions: From age 12 to 14, participants with lower activity facet scores showed higher attrition (OR = 4.21, 95% CI = 1.46, 12.18, *p* = .008), and from age 14 to 16, boys were more likely to drop out than girls (OR = 1.83, 95% CI = 1.03, 3.26, *p* = .040).

### Results

Descriptive results are summarized in the Online Supplemental Material. Table S6 displays means and standard deviations for all Big Five personality traits for ages 10, 12, 14, and 16 for both sexes.

### Personality Trajectories and Sex Differences in Change Across Age

To model overall personality trajectories from age 10 to 16 for girls and boys, latent growth curves (Figure 2) were estimated with sex as the predictor of intercept and growth factors.

**Figure 2.**
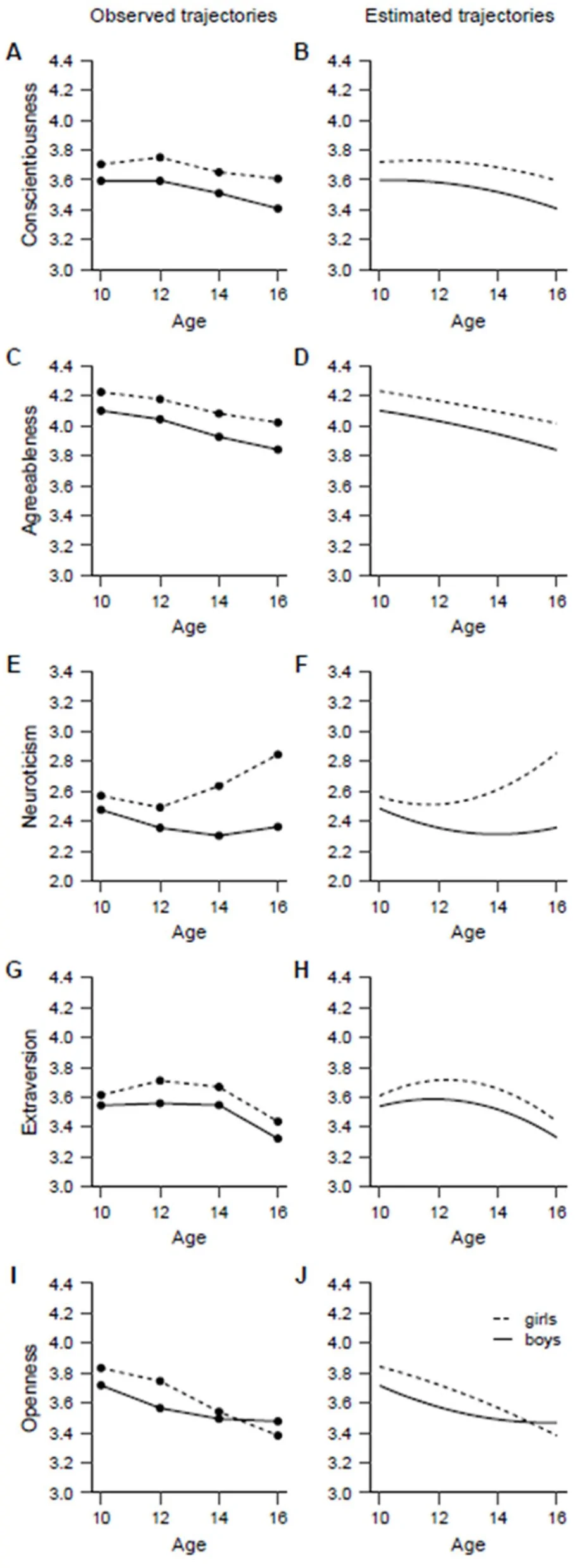
Observed means (Panels A, C, E, G, and I) and estimated trajectories based on latent growth curve models (Panels B, D, F, H, and J) of Big Five personality traits across ages 10 to 16 for girls and boys.

The panels on the left side of Figure 2 graphically present means for all Big Five personality traits for ages 10, 12, 14, and 16, and the corresponding estimates are displayed and described in Online Supplemental Material (Table S8). Intercorrelations of all personality traits at all ages are provided in the Online Supplemental Material in Table S7. Please note that this table also shows associations for each trait across age, thus reflecting the test-retest correlations. As can be seen, the 2-year rank-order stability was moderate across development, with no apparent age differences in stability.

We first modelled linear growth models. As shown in Table 3, model fit was not adequate for any personality traits except for Agreeableness. When adding an additional quadratic slope, the model fit substantially improved and showed indices in accordance with suggested thresholds for all five personality traits. Moreover, χ^2^-difference tests showed that including a quadratic slope significantly improved the model fit for all five personality traits (*p* < .05). The estimated mean growth curves are presented in panels on the right side of Figure 2, and the graphs show trajectories closely following the observed means as presented on the left side of Figure 2. Detailed information about the estimated trajectories is presented in Tables S8 and S9 in the Online Supplemental Material.

**Table 3.** Model Fit for Growth Curve Models with Linear and Curvilinear Trajectories.

BFI traits	χ^2^	*df*	CFI	TLI	RMSEA	90% CI of RMSEA
Linear development (only linear slope estimated)						
Conscientiousness	40.46	7	.944	.920	.077	.055–.101
Agreeableness	18.69	7	.973	.961	.046	.021–.071
Neuroticism	49.28	7	.913	.876	.087	.065–.110
Extraversion	78.22	7	.878	.825	.112	.091–.136
Openness	39.16	7	.945	.921	.076	.054–.099
Curvilinear development (linear and quadratic slope estimated)						
Conscientiousness	5.31	2	.994	.972	.045	.000–.094
Agreeableness	2.06	2	1.000	.999	.006	.000–.070
Neuroticism	1.78	2	1.000	1.002	.000	.000–.067
Extraversion	3.16	2	.998	.990	.027	.000–.080
Openness	3.31	2	.998	.989	.029	.000–.081

*Note. df* = degrees of freedom, CFI = Confirmatory Fit Index, TLI = Tucker-Lewis Index, RMSEA = Root Mean Square Error of Approximation, CI = confidence interval.

To obtain detailed information about changes from one time point to the next for both sexes, we constructed latent change score models for each personality trait with sex as a predictor of change. Because these models were just identified, model fit was not relevant. The results of these analyses are presented in Table 4. Results confirmed trends observed in Figure 2 by showing that conscientiousness and agreeableness did not change significantly from age 10 to 12 for any sex, but decreased significantly thereafter, with one exception (girls’ decrease from age 14 to 16 for conscientiousness did not reach significance). Neuroticism decreased significantly in boys from age 10 to age 12 and thereafter remained stable. In contrast, girls’ neuroticism scores increased substantially from age 12 to 16. Extraversion showed a marked and significant decline from age 14 to 16. Moreover, openness declines significantly among both girls and boys at all ages, besides boys’ change at age 14 to 16.

**Table 4.** Results of Latent Change Score Models for Big Five Personality Traits From Age 10 to 16 for Girls and Boys.

	Latent change from age 10 to 12 (η_2_)			Latent change from age 12 to 14 (η_3_)			Latent change from age 14 to 16 (η_4_)		
BFI traits	Mean	95% CI	*p*	Mean	95% CI	*p*	Mean	95% CI	*p*
Conscientiousness									
Girls	0.046	[−0.016; 0.109]	.148	−0.099	[−0.159; −0.038]	.001	−0.052	[−0.117; 0.013]	.120
Boys	0.001	[−0.070; 0.073]	.927	−0.087	[−0.148; −0.027]	.004	−0.094	[−0.161; −0.028]	.006
Agreeableness									
Girls	−0.048	[−0.105; 0.010]	.103	−0.097	[−0.148; −0.045]	<.001	−0.062	[−0.117; −0.007]	.027
Boys	−0.048	[−0.120; 0.024]	.191	−0.115	[−0.172; −0.058]	<.001	−0.088	[−0.151; −0.026]	.006
Neuroticism									
Girls	−0.075	[−0.150; 0.001]	.052	0.140	[0.062; 0.218]	<.001	0.215	[0.127; 0.303]	<.001
Boys	−0.121	[−0.194; −0.048]	.001	−.059	[−0.131; 0.014]	.112	0.058	[−0.029; 0.144]	.191
Extraversion									
Girls	0.094	[0.033; 0.154]	.002	−0.041	[−0.101; 0.019]	.180	−0.230	[−0.301; −0.159]	<.001
Boys	0.017	[−0.052; 0.086]	.632	−0.018	[−0.086; 0.050]	.605	−0.224	[−0.293; −0.155]	<.001
Openness									
Girls	−0.087	[−0.149; −0.026]	.005	−0.206	[−0.266; −0.146]	<.001	−0.159	[−0.226; −0.093]	<.001
Boys	−0.145	[−0.219; −0.070]	<.001	−0.080	[−0.150; −0.011]	.024	−0.022	[−0.085; 0.041]	.497

*Note*. 95% CI = 95% confidence interval of mean change.

Table 5 displays regression coefficients for the latent change scores on sex, providing information as to whether girls’ increases in personality traits from one time to the next were steeper than boys’ (or whether girls decreased less than boys). Moreover, in this table, we also report information about sex differences in the level of personality traits at age 10 (sex-specific means are displayed in Table S6, online material). Results show significantly higher scores in conscientiousness, agreeableness, and openness among girls at age 10. When examining sex differences in change, girls showed a greater increase in neuroticism than boys from age 12 to 14 and from age 14 to 16. In addition, openness decreased more among girls than boys from age 12 to 14 and age 14 to 16, whereas no other sex differences in change were observed.

**Table 5.** Results of Tests for Sex Differences in the Development of Big Five Personality Traits by Using Latent Change Score Models With Sex as Predictor of Baseline at Age 10 and Change Across Age 10 to 16.

	Sex predicting scores at age 10 (boys = 0, girls = 1)^a^			Sex predicting change from age 10 to 12 (η_2_) (boys = 0, girls = 1)^b^			Sex predicting change from age 12 to 14 (η_3_) (boys = 0, girls = 1)^b^			Sex predicting change from age 14 to 16 (η_4_) (boys = 0, girls = 1)^b^		
BFI traits	*Coef.*	95% CI	*p*	*Coef.*	95% CI	*p*	*Coef.*	95% CI	*p*	*Coef.*	95% CI	*p*
Conscientiousness	0.114	[0.025; 0.202]	.012	0.045	[−0.050; 0.140]	.353	−0.011	[−0.097; 0.074]	.792	0.043	[−0.050; 0.136]	.366
Agreeableness	0.133	[0.056; 0.210]	.001	0.000	[−0.092; 0.092]	.999	0.018	[−0.058; 0.095]	.639	0.027	[−0.056; 0.109]	.530
Neuroticism	0.086	[−0.007; 0.179]	.070	0.046	[−0.058; 0.151]	.384	0.199	[0.093; 0.304]	<.001	0.157	[0.034; 0.280]	.012
Extraversion	0.067	[−0.015; 0.149]	.109	0.077	[−0.015; 0.168]	.099	−0.023	[−0.114; 0.067]	.617	−0.006	[−0.105; 0.093]	.911
Openness	0.118	[0.026; 0.211]	.012	0.058	[−0.039; 0.154]	.242	−0.126	[−0.217; −0.034]	.007	−0.137	[−0.229; −0.045]	.003

*Note.* 95% CI = 95% confidence interval of coefficient.

a Coefficients can be interpreted as how much higher girls scored on the personality trait than boys at age 10.

b Coefficients can be interpreted as how much more girls increased (or how much less girls decreased) than boys in the personality from one time point to the next.

### Development and Sex Differences at the Facet Level

To obtain a more detailed description of the rate and timing of personality changes, we examined developmental patterns of personality at the facet level (Soto & John, 2009). A graphical display of personality facet mean scores according to sex and age is presented in Figure 3. Moreover, Table 6 provides the results of latent change score models. The results show that the overall decrease in conscientiousness between ages 12 and 14 was due to significant decreases in self-discipline, whereas order showed less change across ages. Regarding the facets of agreeableness, altruism showed significant decreases for both sexes from age 14 to 16, whereas compliance decreased significantly from age 12 to 14 for both girls and boys. Neuroticism captures the facets of anxiety and depression, and our results showed that anxiety scores increased significantly in girls from age 12 to 16 but were more stable among boys. Depression scores decreased in boys from age 10 to 14. A similar decrease in depression was observed for girls from age 10 to 12; however, mean scores in depression did not change from age 12 to 14 but increased significantly from age 14 to 16. The two extraversion facets also developed differently across time: Assertiveness scores showed a reversed U-shape for both girls and boys, whereas activity scores declined substantially for both sexes from age 12 to 16. Moreover, the scores of the openness facet aesthetics substantially declined across age for girls, whereas a U-shaped developmental pattern was observed for boys. Openness also subsumes ideas, which remained rather stable for both sexes from age 10 to 12, with a decline at older age for girls and a somewhat lesser decline for boys.

**Figure 3.**
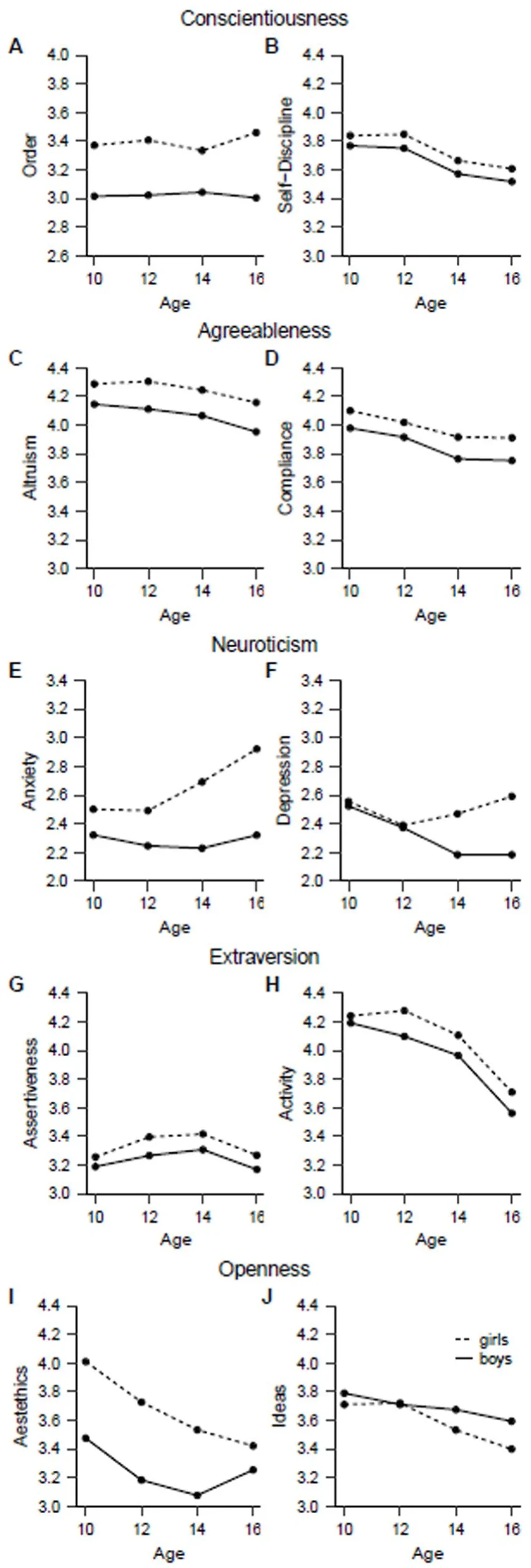
Means of Big Five personality facets across ages 10 to 16 for girls and boys (A-B) Order. (C-D) Agreeableness. (E-F) Neuroticism. (G-H) Extraversion. (I-J) Openness.

**Table 6.** Results of Latent Change Score Models for 10 Facets From Age 10 to 16 for Girls and Boys.

BFI traits and facets	Latent change from age 10 to 12 (η_2_)			Latent change from age 12 to 14 (η_3_)			Latent change from age 14 to 16 (η_4_)		
	Mean	95% CI	*p*	Mean	95% CI	*p*	Mean	95% CI	*p*
Conscientiousness									
Order									
Girls	0.036	[−0.084; 0.155]	.562	−0.073	[−0.180; 0.034]	.184	0.124	[0.012; 0.236]	.030
Boys	0.009	[−0.115; 0.134]	.881	0.020	[−0.086; 0.126]	.716	−0.044	[−0.163; 0.074]	.464
Self-discipline									
Girls	0.009	[−0.063; 0.081]	.811	−0.184	[−0.254; −0.114]	<.001	−0.056	[−0.132; 0.019]	.141
Boys	−0.016	[−0.102; 0.071]	.724	−0.182	[−0.258; −0.107]	<.001	−0.051	[−0.135; 0.032]	.229
Agreeableness									
Altruism									
Girls	0.016	[−0.053; 0.086]	.644	−0.060	[−0.120; 0.000]	.050	−0.087	[−0.158; −0.016]	.017
Boys	−0.034	[−0.122; 0.055]	.455	−0.048	[−0.120; 0.024]	.191	−0.110	[−0.191; −0.029]	.008
Compliance									
Girls	−0.082	[−0.166; 0.002]	.057	−0.101	[−0.178; −0.024]	.010	−0.005	[−0.085; 0.075]	.907
Boys	−0.063	[−0.161; 0.035]	.208	−0.151	[−0.234; −0.068]	<.001	−0.013	[−0.104; 0.079]	.787
Neuroticism									
Anxiety									
Girls	−0.010	[−0.103; 0.084]	.842	0.202	[0.108; 0.295]	<.001	0.229	[0.130; 0.329]	<.001
Boys	−0.076	[−0.170; 0.017]	.109	−0.019	[−0.109; 0.071]	.678	0.091	[0.000; 0.182]	.050
Depression									
Girls	−0.166	[−0.274; −0.058]	.003	0.078	[−0.023; 0.179]	.132	0.123	[0.004; 0.243]	.042
Boys	−0.154	[−0.277; −0.032]	.014	−0.187	[−0.280; −0.094]	<.001	0.002	[−0.121; 0.125]	.970
Extraversion									
Assertiveness									
Girls	0.139	[0.063; 0.214]	<.001	0.019	[−0.052; 0.090]	.602	−0.147	[−0.230; −0.065]	<.001
Boys	0.075	[−0.006; 0.157]	.071	0.040	[−0.044; 0.124]	.353	−0.136	[−0.215; −0.056]	.001
Activity									
Girls	0.035	[−0.044; 0.115]	.380	−0.171	[−0.247; −0.094]	<.001	−0.398	[−0.484; −0.312]	<.001
Boys	−0.094	[−0.199; 0.011]	.078	−0.133	[−0.216; −0.051]	.001	−0.399	[−0.489; −0.309]	<.001
Openness									
Aesthetics									
Girls	−0.284	[−0.396; −0.171]	<.001	−0.195	[−0.313; −0.077]	.001	−0.112	[−0.227; 0.003]	.056
Boys	−0.286	[−0.421; −0.150]	<.001	−0.109	[−0.255; 0.037]	.142	0.180	[0.054; 0.307]	.005
Ideas									
Girls	0.011	[−0.054; 0.077]	.729	−0.188	[−0.256; −0.120]	<.001	−0.133	[−0.205; −0.062]	<.001
Boys	−0.081	[−0.165; 0.003]	.059	−0.035	[−0.108; 0.038]	.351	−0.081	[−0.148; −0.014]	.018

*Note.* 95% CI = 95% confidence interval of mean change.

Finally, Table 7 presents sex differences in facet scores at age 10 and change across age. Girls had significantly higher mean scores at age 10 in order, altruism, compliance, anxiety, and aesthetics. Moreover, girls showed greater increases in the anxiety and depression facets and a greater decrease in ideas than boys from ages 12 to 14. From age 14 to 16, sex differences in order, anxiety, and aesthetics significantly increased.

**Table 7.** Results of Tests for Sex Differences in the Development of 10 Facets by Using Latent Change Score Models With Sex as Predictor of Baseline at Age 10 and Change Across Age 10 to 16.

	Sex predicting scores at age 10 (boys = 0, girls = 1)^a^			Sex predicting change from age 10 to 12 (η_2_) (boys = 0, girls = 1)^b^			Sex predicting change from age 12 to 14 (η_3_) (boys = 0, girls = 1)^b^			Sex predicting change from age 14 to 16 (η_4_) (boys = 0, girls = 1)^b^		
BFI facets	*Coef.*	95% CI	*p*	*Coef.*	95% CI	*p*	*Coef.*	95% CI	*p*	*Coef.*	95% CI	*p*
Order (C)	0.357	[0.207; 0.506]	<.001	0.026	[−0.146; 0.198]	.767	−0.093	[−0.243; 0.058]	.229	0.168	[0.006; 0.330]	.042
Self-discipline (C)	0.071	[−0.034; 0.177]	.186	0.024	[−0.088; 0.137]	.670	−0.002	[−0.105; 0.101]	.972	−0.005	[−0.117; 0.107]	.972
Altruism (A)	0.143	[0.052; 0.233]	.002	0.050	[−0.062; 0.163]	.383	−0.012	[−0.105; 0.082]	.803	0.023	[−0.084; 0.130]	.670
Compliance (A)	0.121	[0.013; 0.229]	.028	−0.019	[−0.148; 0.110]	.771	0.050	[−0.063; 0.163]	.388	0.008	[−0.113; 0.129]	.899
Anxiety (N)	0.178	[0.063; 0.293]	.002	0.067	[−0.064; 0.198]	.317	0.211	[0.091; 0.350]	.001	0.139	[0.005; 0.272]	.043
Depression (N)	0.032	[−0.096; 0.161]	.620	−0.012	[−0.175; 0.152]	.889	0.265	[0.128; 0.402]	<.001	0.121	[−0.050; 0.292]	.165
Assertiveness (E)	0.068	[−0.031; 0.168]	.179	0.063	[−0.048; 0.174]	.263	−0.021	[−0.131; 0.089]	.708	−0.012	[−0.126; 0.103]	.841
Activity (E)	0.051	[−0.062; 0.163]	.377	0.130	[−0.001; 0.261]	.052	−0.037	[−0.149; 0.075]	.514	0.001	[−0.123; 0.126]	.985
Aesthetics (O)	0.541	[0.390; 0.691]	<.001	0.002	[−0.174; 0.178]	.984	−0.085	[−0.273; 0.103]	.373	−0.292	[−0.464; −0.122]	.001
Ideas (O)	−0.080	[−0.177; 0.017]	.108	0.092	[−0.014; 0.198]	.088	−0.153	[−0.253; −0.054]	.003	−0.052	[−0.151; 0.046]	.298

*Note.* 95% CI = 95% confidence interval of coefficient.

a Coefficients can be interpreted as how much higher girls scored on the personality trait than boys at age 10.

b Coefficients can be interpreted as how much more girls increased (or how much less girls decreased) than boys in the personality from one time point to the next.

## Discussion

A better understanding of what characterizes personality development during adolescence may enable us to foster adaptive development of traits known to be important predictors of health, subjective well-being, and good psychosocial functioning (Strickhouser et al., 2017). In the present inquiry, we used four waves of self-reported data from a birth cohort of Norwegian children assessed biennially from age 10 to 16 to examine changes in Big Five personality traits and their facets. In accordance with previous results, conscientiousness and agreeableness dropped, and neuroticism increased, but the latter change was only evident in girls. We add to existing research by showing that for all three traits, the disruption in maturity occurred from age 12 onwards. Extraversion decreased from age 14 to 16, whereas openness decreased at all ages, with one exception. Sex-specific analyses revealed that girls showed a greater increase in neuroticism than boys, in addition to a greater decrease in openness. Hence, the disruption was, in some respects, stronger in girls. The findings further demonstrate that maturation disruptions vary at the level of the facets, indicating a complex developmental process of personality development.

### Conscientiousness Declines from Age 12 in Both Sexes, and Self-Discipline is the Driver

The decline in conscientiousness in early adolescence in our study supports the disruption hypotheses and accords with previous research (Bleidorn et al., 2022; Göllner et al., 2017; Soto et al., 2011; Tackman et al., 2017; Van den Akker et al., 2014), although a substantial number of studies did not report such a decline (de Moor et al., 2023; Jones et al., 2022; Pullmann et al., 2006; Slobodskaya & Kornienko, 2021; Tetzner et al., 2023; Van Dijk et al., 2020). In the present study, we found the decline in conscientiousness to be evident from age 12, not before, whereas Soto et al. (2011) report the decline to start at age 10 (i.e., their youngest age group), and Tackman et al. (2017) reported a decrease from age 10 to 13, followed by an increase from age 13 to 15. Because assessments were made only at ages 10 and 13 in the latter study, the decline might have happened any time in between, for example, at age 12, as found in the present inquiry. Using annual assessments and estimating growth curves, Van den Akker et al. (2014) found the decline to be evident from age 9 to 15, but it was not explicitly tested whether the decrease was evident at specific ages. Thus, although existing studies suggest that the decline starts earlier than revealed here, they fail to test the exact timing of the decline. Although we add by using 2-year measurement intervals, future studies should aim to examine shorter time spans to reveal the exact timing of the drop in conscientiousness, also taking into account that the development may vary between cultures, ethnicities, and countries. Moreover, to account for potential period and cohort effects, future studies should perform age-period-cohort analyses to separate age effects from effects due to period or cohort.

Finally, our facet analyses indicated that the decline in overall conscientiousness was due to decreases in self-discipline, a change that did not differ between the sexes. It is not obvious why self-discipline and not order accounts for the decline in conscientiousness, but it might be related to how the two facets were measured. Order was only captured by two quite broad items (i.e., ‘can be somewhat careless’, ‘tends to be disorganized’), whereas self-discipline was assessed by five more specific items (e.g., ‘makes plans and follows through with them’). The latter facet may thus be better positioned to capture the changes adolescents experience, and which may be related to the increase in reward sensitivity and still developing cognitive control characterizing the adolescent brain, as detailed in the Introduction.

### Agreeableness Declines After Age 12 in Both Sexes, and the Drivers are Age-Dependent

We found the same pattern for agreeableness as for conscientiousness: a decline from age 12 onwards in both girls and boys. Previous research (Göllner et al., 2017; Slobodskaya & Kornienko, 2021; Soto et al., 2011; Van den Akker et al., 2014) also generally supports the disruption hypotheses (Tetzner et al., 2023). Yet again, existing findings suggest that the dip occurs at a younger age than we found. In line with previous studies (Göllner et al., 2017; Slobodskaya & Kornienko, 2021; Tetzner et al., 2023; Van den Akker et al., 2014), we found girls to display higher levels of agreeableness, although the development of agreeableness was not sex-specific. Different facets seem to drive the decrease in agreeableness at different ages: from age 12 to 14 years, compliance significantly decreased; from age 14 to 16 years, altruism significantly decreased. This accords with and refines former findings of parent-reported facets showing changes in compliance and altruism to be evident from middle childhood to the end of middle adolescence (de Haan et al., 2017). Compliance, which reflects whether adolescents perceive themselves as quarrelsome, may decrease more during the transition to adolescence (ages 12–14) when the need for autonomy and detachment from parents begins to rise. Furthermore, with increasing age, adolescents may become more realistic in their self-assessment, especially in social behavior, which may explain why altruism (e.g., ‘is helpful and unselfish with others’) did not decline until age 14.

### Girls’ Level of Neuroticism Increases, Driven by the Anxiety Facet

In accordance with two previous studies of self-reported personality (Soto et al., 2011; Van den Akker et al., 2014), we found neuroticism to increase in girls, but not boys, from age 12 to 16. Göllner et al. (2017), on the other hand, report neuroticism to be stable from age 11 to 14. In Soto et al.’s (2011) study, the decline was evident from the youngest age group onwards (age 10), and Van den Akker et al. (2014) reported girls to increase more than boys from age 9 to 17, but the exact timing of the increase was not examined in the latter study. Thus, yet again, we found the disruption to appear at a later age than in previous studies. However, neither previous nor the current research can identify the exact timing of change, which would require more granular assessments. At the facet level, anxiety scores significantly increased in girls from age 12 to 16 but were more stable among boys, thus driving the overall comparative increase in neuroticism in girls. The depression facet, on the other hand, decreased in girls from age 10 to 12, then remained stable from age 12 to 14 before increasing from age 14 to 16. Boys showed a decrease in depression from age 10 to 14, and no change from age 14 to 16. Thus, the anxiety facet scores seem to play a major role in explaining girls’ increase in neuroticism (Brandes et al., 2021; de Haan et al., 2017). Two potential explanations might shed light on why girls display increased neuroticism in the present study, whereas boys do not: girls may be either (a) more vulnerable to factors enhancing neuroticism or (b) more exposed to them. Regarding the former, stressful life events have been found to predict higher levels of neuroticism in adolescence (Goldstein et al., 2020), and females might face more adversities in response to stress than do males (Rincon-Cortes et al., 2019). Women are also found to be more vulnerable to interpersonal stress (Shih et al., 2006), and interpersonal conflicts are known to predict increased neuroticism (Borghuis et al., 2020). Moreover, girls are more *exposed* to victimization and social exclusion than boys (Hankin et al., 2015), which could further contribute to their elevated neuroticism. Furthermore, sex hormones have been implicated in explaining why women are more vulnerable to anxiety, stress, and trauma (Li & Graham, 2017). However, Van den Akker et al. (2021) found neuroticism to be unrelated to both pubertal hormones and self-reported puberty in girls.

### Openness Declines From Age 10 in Both Sexes

Consistent with the findings of Soto et al. (2011), we observed a decline in openness from age 10 onwards. Of note, between ages 12 to 14 and 14 to 16, the decrease in openness was greater among girls than boys. The greater decline in girls was mirrored in their substantial decrease in the aesthetics facet, whereas a U-shaped developmental pattern was observed for boys. The ideas facet remained rather stable for both sexes from age 10 to 12, with a subsequent decline at older ages among girls and yet again a somewhat lesser decline for boys. Social role theories (Eagly, 1987) may offer insights into why girls experienced a more substantial decrease in openness compared to boys. In line with Western gender role expectations and socialization, characteristics associated with high levels of openness—such as creativity, intellectuality, openness to ideas—are more closely related to the male gender ideal, whereas the female ideal is more closely associated with conformity. However, research has failed to find evidence for the basic assumption of social role theories, which posits that sex differences in personality should be smaller in gender-egalitarian societies (De Bolle et al., 2015). The larger decline in openness among girls could also stem from sex differences in self-reporting. Given that openness is positively valued in a Western individualistic cultures, and considering that adolescent girls display lower self-esteem than boys (von Soest et al., 2016), girls might be more likely to underrate their openness (e.g., ‘I am not creative’), whereas boys might be prone to overrate it (e.g., ‘I am full of ideas’).

### Extraversion Declines from Age 14 to 16 in Both Sexes, and the Driver is Activity

Extraversion scores were consistently higher for girls than boys at all time points except at age 10, which accords with findings from a German school sample (Göllner et al., 2017). No change in extraversion was found until it significantly dropped in both sexes from age 14 to 16. At the facet level, activity showed a substantial decline from age 12 onwards in both sexes, which corresponds to the findings by Soto et al. (2011), who reported a marked decline in activity from late childhood through adolescence. From a developmental perspective, this decline makes sense, as the activity facet captures energy and enthusiasm—traits more commonly seen in childhood before the influence of social comparisons and stricter social rules associated with adolescence diminishes such characteristics. Additionally, as adolescents grow older, they tend to channel more of their activity into social, work, and school settings. As further found both in the present study and the seminal work by Soto et al. (2011), assertiveness showed a bell-shaped development for both sexes. Assertiveness, which encompasses social dominance, talkativeness, and expressiveness, initially increased, likely due to the importance of these characteristics in gaining or maintaining a social position.

### Summary and Implications of Findings

Overall, our results support the disruption hypothesis as declines were evident in conscientiousness and agreeableness for both sexes, whereas neuroticism increased in girls only. For some traits, the dip was driven by one facet; for others, both facets contributed. This indicates that personality development is more complex at the facet level, which has also been found in parent- and teacher-reported studies (Brandes et al., 2021; de Haan et al., 2017; Prinzie & Dekovic, 2008) and in studies of older adolescents and young adults (Ringwald et al., 2024). We found the dip in maturation to be evident from the second assessment point when the participants had a mean age of 12.5 years. Norwegian students transition from primary school to middle school (junior high) in the year they turn 13, when they encounter new teachers, schoolmates, and subjects, as well as graded exams for the first time. This timing aligns with the Social Investment Theory, which states that new developmental tasks and demands, such as increased school requirements or a new social setting, for which an individual has not yet developed the capabilities to handle, can result in a temporary drop in personality maturity (Roberts et al., 2005).

As expected, boys display lower levels of conscientiousness and agreeableness than girls at all time points. These findings may indicate that boys and girls need different kinds of support to develop adaptive personality traits. Interventions aimed to promote personality traits related to beneficial outcomes (Javaras et al., 2019) should likely be sex-specific. For example, as it has been shown that conscientiousness mediates the sex gap in school achievement (with girls outperforming boys) (Verbree et al., 2022), promoting conscientiousness in boys may help their school achievements and reduce this gap.

Girls were at increased risk of developing higher levels of neuroticism in the transition to adolescence. Because this increase corresponds with the increase in emotional disorders in girls during adolescence (Salk et al., 2017) and research suggests that neuroticism is a transdiagnostic risk factor for psychopathology (Lynch et al., 2021), being bidirectionally related to depression (Morken et al., 2024), targeted efforts should be made to prevent such development. Although evidence is still preliminary, studies suggest that psychoeducation, stress management interventions, and strategies to promote educational emotional acceptance and reduce stress in response to strong emotions can decrease neuroticism (Allemand & Fluckiger, 2022; Armstrong & Rimes, 2016; Farchione et al., 2012; Hudson & Fraley, 2015; Sauer-Zavala et al., 2017).

### Limitations

The present findings should be interpreted in light of several limitations. First, although we argue for the importance of using self-reports—since parents become less positioned to reliably report on their offspring’s behavior, thoughts, and feelings from adolescence onwards—younger children are generally less reliable reporters than older adolescents, as indicated by the internal consistency of the BFI increasing by age. Consistent with previous research (Soto et al., 2011), internal consistency at age 10 was low, particularly for facets with few indicators, which may suggest that the traits and facets were not reliably assessed before age 12. However, validation studies of the BFI and other short personality scales have demonstrated that measures with low internal consistency can still exhibit high reliability through other metrics. For example, Soto and John (2009) found that the two-item depression facet of the BFI had an internal consistency of only α = .53, yet its stability was high, with a test-retest reliability of *r* = .82. Similarly, a meta-analysis of the reliability of the Ten-Item Personality Inventory showed that, although internal reliabilities for all two-item trait measures were below α = .60, test-retest reliabilities indicated substantial stability, with estimates ranging from *r* = .69 to *r* = .78 (Thorrisen & Sadeghi, 2023). In the present study, the stabilities from age 10 to 12 were of the same magnitude as the subsequent 2-year stabilities. Therefore, low internal consistency in measures with few items does not necessarily indicate poor reliability when other metrics, such as test-retest correlations, are taken into account. Nonetheless, the particular low internal consistency in younger age groups remains a concern, particularly because individual differences in acquiescence (i.e., the tendency to consistently agree or disagree) are more pronounced in younger age groups (Soto et al., 2011), potentially confounding mean-level differences across ages.

Confirmatory factor analyses showed that all traits and facets showed acceptable metric invariance across the four data collection waves. However, while some traits and facets showed an acceptable model fit under scalar invariance, others displayed suboptimal fit, with the compliance facet showing particularly poor fit. These challenges with scalar invariance suggest that the interpretation of the BFI items may change from age 10 to age 16. Future cohort studies of personality development should, therefore, include both self-reports and reports from parents or peers to provide a more comprehensive understanding of whether personality age trends in young people are replicable when using observer reports.

Nearly 95% of the participants were of ethnic Norwegian origin. This raises concerns about the generalizability of our findings to other ethnic groups or cultures, given that cultural differences in perceptions of age-appropriate behavior and social role expectations may affect personality development (Ni et al., 2023), as discussed in the context of Social Investment Theory. This limitation, as noted by Bleidorn et al (2022), is common to most longitudinal studies of personality development. Notably, some non-Western studies support the assumption of cultural differences in personality development. For example, a mean level increase in conscientiousness was observed in Chinese youth from ages 8 to 14 (Ni et al., 2023), while conscientiousness remained stable from age 11 to 17 in a Russian study (Slobodskaya & Kornienko, 2021). Moreover, although our sample is comparable to the Norwegian population in terms of parental education levels (Statistics Norway, 2012) and family situations (i.e., parents married, one-parent household, etc.) (Statistics Norway, 2017), the Norwegian population is generally more well-educated, and experiences smaller social inequalities compared to many other countries (Eurostat, 2023; Mathiesen, 2023). Since parental socioeconomic status, although weakly, is associated with offspring personality (Ayoub et al., 2018), these population characteristics may further limit the generalizability of our findings. On the other hand, it has been shown that while cross-sectional associations exist, socioeconomic status is not related to *changes* in adolescent personality (Li et al., 2023).

Finally, although our sample is large, it may still not be powered to detect small changes in personality traits. As reported in previous research, changes identified are typically small (e.g., Göllner et al., 2017; Van den Akker et al., 2014). Our study also comes with the strength of relying on four waves of biennially collected data, but to provide further granularity of the developmental processes at play, future studies examining shorter time intervals (e.g., 1 year, 6 months) are needed.

## Conclusion

In our population-based, longitudinal study of Norwegian adolescents assessed biennially from age 10 to 16 years, we demonstrate that this developmental stage is marked by significant changes in personality traits. Specifically, we observed a disruption of personality development, characterized by declines in conscientiousness and agreeableness, alongside an increase in neuroticism among girls. Our research reveals that these disruptions begin to manifest around the age of 12 and continue thereafter, with certain facets playing a key role in personality development. Future research should focus on exploring the underlying mechanisms driving these changes. To achieve this, there is a need for studies that integrate research on various potential explanatory factors, encompassing social (e.g., peer relations), emotional (e.g., stressful life events), cognitive (e.g., inhibition), and biological (e.g., puberty) aspects. This line of inquiry has the potential to guide the development of targeted interventions aimed at fostering healthy personality growth in adolescents.

## Supplemental Material

sj-docx-1-psp-10.1177_01461672251396595 – Supplemental material for Personality From Age 10 to 16 years. A Four-Wave Cohort Study of Development and Sex Differences in the Big Five and Its Facets Supplemental material, sj-docx-1-psp-10.1177_01461672251396595 for Personality From Age 10 to 16 years. A Four-Wave Cohort Study of Development and Sex Differences in the Big Five and Its Facets by Silje Steinsbekk, Lars Wichstrøm and Tilmann von Soest in Personality and Social Psychology Bulletin

## Data Availability

Data are not available due to restrictions related to participant consent and because the study is still ongoing.

## Notes

1. One former study has used some of the personality data published here [AsPredicted], but at the time of our preregistration, the analysis of that study had not been conducted, and the data had not been explored.
