# Supplementary material for: Personality From Age 10 to 16 years. A Four-Wave Cohort Study of Development and Sex Differences in the Big Five and Its Facets
Source: Pers Soc Psychol Bull. 2025 Dec 26;52(12):4097–116. doi: 10.1177/01461672251396595 (PMC13736290; doi:10.1177/01461672251396595)
Supplement: Supplementary file 1 [file sj-docx-1-psp-10.1177_01461672251396595.docx]

**Online Supplemental Material**

**Deviations from Preregistration**

***Table S1****.* *Preregistration Deviations Table*

| # | Details | | Original Wording | Deviation Description | Reader Impact |
| --- | --- | --- | --- | --- | --- |
| 1 | Type | Other (Please Explain) | Author(s)  Silje Steinsbekk (Norwegian University of Science and Technology) - silje.steinsbekk@ntnu.no  Lars Wichstrøm (Norwegian University of Science and Technology) - lars.wichstrom@ntnu.no  Oliver John (UC Berkeley) - o_johnx5@berkeley.edu  Tilmann von Soest (University of Oslo) - t.v.soest@psykologi.uio.no  Silje Steinsbekk (Norwegian University of Science and Technology) - silje.steinsbekk@gmail.com | Oliver John is not an author of the paper, although he was originally supposed to be. | This deviation has no impact for the reader |
|  | Reason | Other (Please Explain) |  |  |  |
|  | Timing | After data access |  |  |  |
| 2 | Type | Research Q(s) | 2) What's the main question being asked or hypothesis being tested in this study?  a) How do self-reports of the Big Five personality traits develop from age 10 to 16 years in terms of mean levels in a longitudinal sample in Norway? Of particular interest is whether conscientiousness and agreeableness decrease from age 10 to 14 (as shown in the U.S (Soto et al., 2011)), and whether any such decreases are linear or accelerate after age 12. The inclusion of age 16 allows us to test whether any potential decrease continues in later adolescence or whether the development of these trends have reached the lowest points.  b) When do sex differences emerge? For example, do the sex differences in agreeableness already exist by age 10? Do sex differences in neuroticism emerge (or increase) after age 10? Do girls show the hypothesized drop in conscientious earlier than boys?  c) How do these change patterns manifest at the level of the two BFI facets developed by Soto & John (2009)? For example, do they simply mirror the patterns shown by their superordinate domain, or are there meaningful differences (e.g., on extraversion, does activity drop from 10 to 14 whereas assertiveness does not)? | (1) How do self-reported Big Five personality traits and their facets develop from age 10 to 16 years? (2) When do sex differences in developmental patterns emerge? Based on previous research, we are particularly interested in whether overall and sex-specific disruptions in the development of conscientiousness, agreeableness, and neuroticism are replicated. | This deviation has no impact for the reader, as the content is kept although the wording is slightly changed. |
|  | Reason | Peer review |  |  |  |
|  | Timing | After results known |  |  |  |
|  | Reason | Other (Please Explain) |  |  |  |
|  | Timing | After data access |  |  |  |
| **Unregistered Steps** | | | | | |
| # | Details | | Original Wording | Unregistered Step Description | Reader Impact |
| 1 | Type | Analysis |  | Based on the peer review, measurement invariance testing was conducted. |  |
|  | Timing | After results known |  |  |  |
|  | Timing | Select One |  |  |  |

*Note*. The template of this table is developed by Willroth & Atherton (2024)

**Examination of Measurement Invariance Across Age**

To assess measurement invariance in trait measures of the Big Five Inventory (BFI) across age, we conducted a series of confirmatory factor analyses (CFA). Model fit was evaluated by inspecting χ^2^ statistics, the Comparative Fit Index (CFI), the Tucker–Lewis Index (TLI), and the root mean square error of approximation (RMSEA). In line with validation studies of short personality scales (e.g., Borghuis et al., 2017; de Moor et al., 2023), we constructed three parcels for each personality trait. Reducing indicators through parceling has been recommended as it offers more robust tests of structural model parameters by more precisely defining constructs (Little, 2013; Little et al., 2013). Items were assigned to parcels by using the item-to-construct balance technique (Little et al., 2002). Parcels were constructed in an identical manner across all four time points.

Following suggestions by Widaman, Ferrer, and Conger (2010), we first tested for configural invariance across time points by modelling the personality trait in question simultaneously at each wave. In this model, correlations between factors across time points were modelled and error variances of identical items at different time points were allowed to correlate. As shown in Table S2, results show acceptable model fits for all five personality traits.

Next, we tested for metric invariance by comparing the configural invariance model with a model in which factor loadings were constrained to be equal across time points. Differences in fit between the models were assessed using the Satorra-Bentler scaled χ^2^-difference tests. Results showed statistically significant differences when comparing the configural invariant with the metric invariant models for all personality traits except openness (see last columns, Table S2). However, given the relatively large sample size, even small differences in fit may reach statistically significant results. Therefore, we also inspected model fit indices less sensitive to sample size. As shown in Table S2, the CFI, TLI, and RMSEA indicated only minor differences between the configural and the metric invariant models, supporting metric variance for all five personality traits. The change in these indices were consistent with methodological guidelines, which recommend that a decline of no more than .01 in the CFI is acceptable (Cheung & Rensvold, 2002).

Finally, scalar invariance was tested by additionally constraining item intercepts to be equal across time. Results showed statistically significant differences between the metric and scalar invariant models for all personality traits. Moreover, other fit indices (CLI, TLI, RMSEA) indicated a notable decline in model fit, in most cases exceeding the thresholds

***Table S2.*** *Fit for Measurement Models with Varying Degree of Invariance Across Time Points for the Big Five Personality Traits*

|  | χ^2^ | *df* | CFI | TLI | RMSEA | 90% CI of RMSEA | Model Comparison | | | | |
| --- | --- | --- | --- | --- | --- | --- | --- | --- | --- | --- | --- |
|  |  |  |  |  |  |  | Δχ^2^ | Δ*df* | | *p* | |
| Conscientiousness | | | | | | | | | | | |
| Configural invariance | 48.95 | 30 | .992 | .982 | .028 | .012 – .042 |  | |  | |  |
| Metric invariance | 68.31 | 36 | .986 | .974 | .033 | .021 – .045 | 19.36 | | 6 | | .004 |
| Scalar invariance | 146.28 | 45 | .956 | .935 | .053 | .043 – .063 | 79.23 | | 9 | | <.001 |
| Agreeableness | | | | | | | | | | | |
| Configural invariance | 44.91 | 30 | .991 | .980 | .025 | .006 – .043 |  | |  | |  |
| Metric invariance | 49.33 | 36 | .992 | .985 | .021 | .000 – .035 | 4.71 | | 6 | | .581 |
| Scalar invariance | 199.99 | 45 | .904 | .860 | .065 | .056 – .075 | 149.79 | | 9 | | <.001 |
| Neuroticism | | | | | | | | | | | |
| Configural invariance | 33.38 | 30 | .998 | .997 | .012 | .000 – .030 |  | |  | |  |
| Metric invariance | 50.87 | 36 | .993 | .987 | .023 | .000 – .036 | 16.73 | | 6 | | .010 |
| Scalar invariance | 142.20 | 45 | .955 | .934 | .052 | .042 – .062 | 95.27 | | 9 | | <.001 |
| Extraversion | | | | | | | | | | | |
| Configural invariance | 47.50 | 30 | .992 | .981 | .027 | .010 – .041 |  | |  | |  |
| Metric invariance | 60.95 | 36 | .988 | .978 | .029 | .016 – .042 | 13.20 | | 6 | | .040 |
| Scalar invariance | 277.76 | 45 | .888 | .836 | .080 | .071 – .089 | 216.61 | | 9 | | <.001 |
| Openness | | | | | | | | | | | |
| Configural invariance | 31.75 | 30 | .999 | .998 | .009 | .000 – .028 |  | |  | |  |
| Metric invariance | 36.96 | 36 | .999 | .999 | .006 | .000 – .026 | 5.26 | | 6 | | .511 |
| Scalar invariance | 228.34 | 45 | .899 | .852 | .071 | .062 – .080 | 194.38 | | 9 | | <.001 |

Notes. CFI = Comparative Fit Index; TLI = Tucker-Lewis Index; RMSEA = Root Mean Square Error of Approximation; CI = Confidence Interval. Satorra-Bentler

scaled χ^2^-test were conducted when comparing models

suggested by Cheung & Rensvold (2002). While the scalar invariance models for conscientiousness and neuroticism demonstrated acceptable fit, some fit indices for the remaining three traits fell below commonly accepted cutoffs. We therefore examined partially metric invariant models, where we freed up metric constraints guided by modification indices. For agreeableness, freeing up three metric constraints (one at age 10, one at age 14, and one at age 16) resulted in a good fit: χ^2^(42) = 88.01; CFI = .972; TLI = .955; RMSEA = .037; 95% *CI* = [.026–.048]. Likewise, freeing up three metric constraints (one at age 14 and two at age 16) provided good fit: χ^2^(42) = 92.26; CFI = .976; TLI = .962; RMSEA = .039; 95% *CI* = [.028–.049]. Finally, also for openness, freeing up three metric constraints (one at age 10, one at age 14, and one at age 16) provided a good solution: χ^2^(42) = 86.36; CFI = .976; TLI = .962; RMSEA = .036; 95% *CI* = [.025–.047].

In conclusion, the results indicate metric invariance for all five personality traits. However, model fit indices suggest only partial metric invariance for agreeableness, extraversion, and openness. In a previous study where we tested for measurement invariance of the BFI we adjusted for acquiescence bias (Morken et al., 2024), as recommended by Soto et al. (2008), but such adjustments this did not substantially change model fit.

We applied the same analytical framework to examine measurement invariance for the facets of the BFI. Again, three parcels were constructed for each facet at all four time points. For facets consisting of only three items, the individual items were used as the basis for CFA instead of parcels. Invariance tests for the three facets that contained only two items (Activity, Order, and Depression) were not conducted, as the measurement models were not identifiable.

As shown in Table S3, the results indicate an acceptable model fit for both configural and metric invariance across all seven facets investigated. For assertiveness, altruism, and compliance, model fit did not significantly deteriorate when comparing the metric invariant models to the configural invariant models. For the remaining four facets, a significant decline in model fit was observed as assessed by Satorra-Bentler scaled χ^2^-difference tests. However, despite this decline, other fit indices showed only very modest reductions in model fit (Cheung & Rensvold, 2002), and the metric invariance models still exhibited appropriate fit.

When testing for scalar invariance, model fit significantly declined for all seven models. Although some models, such as those for assertiveness and anxiety, maintained adequate fit, low values—particularly for the TLI— were found in several models. The compliance facet in particular showed suboptimal fit. When testing for partial scalar invariance for this facet we achieved a good model fit by freeing up four intercepts (one at age 10, one at age 12, and two at age 16): χ^2^(42) = 51.68; CFI = .981; TLI = .970; RMSEA = .018; 95% *CI* = [.000–.032].

In summary, Table S3 shows satisfactory metric invariance for all seven personality facets. However, the substantially reduced model fit for several facets highlights challenges with scalar invariance in some cases.

***Table S3.*** *Fit for Measurement Models with Varying Degree of Invariance Across Time Points for Big Five Personality Facets*

|  | χ^2^ | *df* | CFI | TLI | RMSEA | 90% CI of RMSEA | Model Comparison | | | | |
| --- | --- | --- | --- | --- | --- | --- | --- | --- | --- | --- | --- |
|  |  |  |  |  |  |  | Δχ^2^ | Δ*df* | | *p* | |
| Assertiveness | | | | | | | | | | | |
| Configural invariance | 30.93 | 30 | .999 | .999 | .006 | .000 – .028 |  | |  | |  |
| Metric invariance | 38.26 | 36 | .999 | .997 | .009 | .000 – .027 | 7.34 | | 6 | | .291 |
| Scalar invariance | 106.22 | 45 | .959 | .941 | .041 | .031 – .051 | 69.61 | | 9 | | <.001 |
| Altruism | | | | | | | | | | | |
| Configural invariance | 24.84 | 30 | 1.000 | 1.000 | .000 | .000 – .020 |  | |  | |  |
| Metric invariance | 34.04 | 36 | 1.000 | 1.000 | .000 | .000 – .023 | 9.13 | | 6 | | .166 |
| Scalar invariance | 132.66 | 45 | .899 | .852 | .049 | .040 – .059 | 85.44 | | 9 | | <.001 |
| Compliance | | | | | | | | | | | |
| Configural invariance | 33.97 | 30 | .993 | .985 | .013 | .000 – .031 |  | |  | |  |
| Metric invariance | 41.38 | 36 | .991 | .983 | .014 | .000 – .030 | 7.30 | | 6 | | .294 |
| Scalar invariance | 207.52 | 45 | .717 | .584 | .067 | .058 – .076 | 163.75 | | 9 | | <.001 |
| Self-discipline | | | | | | | | | | | |
| Configural invariance | 30.18 | 30 | 1.000 | 1.000 | .003 | .000 – .027 |  | |  | |  |
| Metric invariance | 50.00 | 36 | .988 | .977 | .022 | .000 – .036 | 19.68 | | 6 | | .003 |
| Scalar invariance | 145.38 | 45 | .911 | .869 | .053 | .043 – .062 | 96.10 | | 9 | | <.001 |
| Anxiety | | | | | | | | | | | |
| Configural invariance | 26.49 | 30 | 1.000 | 1.000 | .000 | .000 – .022 |  | |  | |  |
| Metric invariance | 35.17 | 36 | 1.000 | 1.000 | .000 | .000 – .024 | 19.68 | | 6 | | .003 |
| Scalar invariance | 134.88 | 45 | .944 | .917 | .050 | .040 – .060 | 96.10 | | 9 | | <.001 |
| Aesthetics | | | | | | | | | | | |
| Configural invariance | 46.40 | 30 | .992 | .982 | .026 | .009 – .040 |  | |  | |  |
| Metric invariance | 59.81 | 36 | .988 | .978 | .029 | .015 – .041 | 13.17 | | 6 | | .040 |
| Scalar invariance | 182.91 | 45 | .930 | .897 | .062 | .053 – .071 | 96.68 | | 9 | | <.001 |
| Ideas | | | | | | | | | | | |
| Configural invariance | 38.67 | 30 | .993 | .984 | .019 | .000 – .035 |  | |  | |  |
| Metric invariance | 46.38 | 36 | .991 | .984 | .019 | .000 – .033 | 7.711 | | 6 | | .260 |
| Scalar invariance | 136.70 | 45 | .925 | .890 | .050 | .041 – .060 | 90.64 | | 9 | | <.001 |

*Note*. CFI = Comparative Fit Index; TLI = Tucker-Lewis Index; RMSEA = Root Mean Square Error of Approximation; CI = Confidence Interval. Satorra-Bentler scaled χ^2^-test were conducted when comparing models. Measurement invariance for the facets Activity, Order, and Depression could not be evaluated because they consisted of only two items.

**Examination of Measurement Invariance Across Sex**

As an additional set of analyses, we examined measurement invariance across sex. To this end, we constructed correlated latent factor models across all four time points for each personality trait separately. As in previous models, error variances of identical items at different time points were allowed to correlate. We then specified a two-group model by sex and tested for configural, metric, and scalar invariance across groups. As shown in Table S4, results indicate excellent model fits for all five personality traits at the configural level.

Next, we tested for metric invariance across sex by comparing the configural invariance model with a model in which factor loadings were constrained to be equal across girls and boys. Satorra-Bentler scaled χ^2^-difference tests revealed non-significant changes in model fit for all five personality traits, supporting metric invariance (see Table S4).

We then tested scalar invariance by additionally constraining item intercepts to be equal across sex. Results showed statistically significant differences between the metric and scalar invariance models for all traits except agreeableness. However, the declines in fit indices were minor for all personality traits except openness. Thus, the scalar invariance results support invariance across sex for conscientiousness, agreeableness, neuroticism, and extraversion, while the substantially decline in model fit for openness indicates challenges in establishing scalar invariance for this trait.

We also tested for measurement invariance for the facet scales, which yielded very good model fits for the configural invariance models across all seven facets (see Table S5). When examining metric invariance, four of seven facets showed non-significant decreases in model fit, while the remaining three facets exhibited only moderate decreases, providing overall support for metric invariance. Model fit indices also largely supported scalar invariance, as reductions in fit relative to the metric models were moderate across most indices for all seven facets.

***Table S4.*** *Fit for Measurement Models with Varying Degree of Invariance Across Sex for the Big Five Personality Traits*

|  | χ^2^ | *df* | CFI | TIL | RMSEA | 90% CI of RMSEA | Model Comparison | | |
| --- | --- | --- | --- | --- | --- | --- | --- | --- | --- |
|  |  |  |  |  |  |  | Δχ^2^ | Δ*df* | *p* |
| Conscientiousness | | | | | | | | | |
| Configural invariance | 93.75 | 60 | .985 | .968 | .037 | .022 – .052 |  |  |  |
| Metric invariance | 100.44 | 68 | .986 | .973 | .034 | .019 – .048 | 7.14 | 8 | .521 |
| Scalar invariance | 137.08 | 76 | .973 | .954 | .045 | .032 – .057 | 36.55 | 8 | <.001 |
| Agreeableness | | | | | | | | | |
| Configural invariance | 69.31 | 60 | .994 | .987 | .020 | .000 – .038 |  |  |  |
| Metric invariance | 80.51 | 68 | .992 | .985 | .021 | .000 – .038 | 11.09 | 8 | .197 |
| Scalar invariance | 94.02 | 76 | .989 | .981 | .024 | .000 – .039 | 13.42 | 8 | .098 |
| Neuroticism | | | | | | | | | |
| Configural invariance | 61.62 | 60 | .999 | .998 | .008 | .000 – .032 |  |  |  |
| Metric invariance | 67.39 | 68 | 1.000 | 1.000 | .000 | .000 – .029 | 6.09 | 8 | .637 |
| Scalar invariance | 83.63 | 76 | .996 | .994 | .016 | .000 – .033 | 16.58 | 8 | .035 |
| Extraversion | | | | | | | | | |
| Configural invariance | 72.08 | 60 | .994 | .987 | .022 | .000 – .039 |  |  |  |
| Metric invariance | 84.95 | 68 | .992 | .984 | .025 | .000 – .040 | 12.70 | 8 | .122 |
| Scalar invariance | 120.51 | 76 | .979 | .963 | .038 | .025 – .051 | 36.33 | 8 | <.001 |
| Openness | | | | | | | | | |
| Configural invariance | 77.95 | 60 | .990 | .979 | .027 | .000 – .043 |  |  |  |
| Metric invariance | 87.32 | 68 | .989 | .980 | .027 | .000 – .042 | 9.43 | 8 | .308 |
| Scalar invariance | 190.95 | 76 | .937 | .891 | .061 | .051 – .072 | 113.41 | 8 | <.001 |

Notes. CFI = Comparative Fit Index; TLI = Tucker-Lewis Index; RMSEA = Root Mean Square Error of Approximation; CI = Confidence Interval. Satorra-Bentler

scaled χ^2^-test were conducted when comparing models

***Table S5.*** *Fit for Measurement Models with Varying Degree of Invariance Across Sex for Big Five Personality Facets*

|  | χ^2^ | *df* | CFI | TLI | RMSEA | 90% CI of RMSEA | Model Comparison | | | | |  |
| --- | --- | --- | --- | --- | --- | --- | --- | --- | --- | --- | --- | --- |
|  |  |  |  |  |  |  | Δχ^2^ | Δ*df* | | *p* | | |
| Assertiveness | | | | | | | | | | | |  |
| Configural invariance | 60.59 | 60 | 1.000 | .999 | .005 | .000 – .031 |  | |  |  |  |  |
| Metric invariance | 74.16 | 68 | .996 | .992 | .015 | .000 – .034 | 13.42 | | 8 | .098 |  |  |
| Scalar invariance | 146.95 | 76 | .953 | .918 | .048 | .036 – .060 | 82.98 | | 8 | <.001 |  |  |
| Altruism | | | | | | | | | | | |  |
| Configural invariance | 61.86 | 60 | .998 | .995 | .009 | .000 – .032 |  | |  |  |  |  |
| Metric invariance | 76.40 | 68 | .990 | .981 | .018 | .000 – .035 | 13.75 | | 8 | .089 |  |  |
| Scalar invariance | 92.12 | 76 | .981 | .967 | .023 | .000 – .038 | 15.58 | | 8 | .049 |  |  |
| Compliance | | | | | | | | | | | |  |
| Configural invariance | 72.78 | 60 | .978 | .952 | .023 | .000 – .040 |  | |  |  |  |  |
| Metric invariance | 79.52 | 68 | .980 | .962 | .021 | .000 – .037 | 7.50 | | 8 | .484 |  |  |
| Scalar invariance | 100.28 | 76 | .959 | .928 | .028 | .009 – .042 | 20.73 | | 8 | .008 |  |  |
| Self-discipline | | | | | | | | | | | |  |
| Configural invariance | 60.55 | 60 | 1.000 | .999 | .005 | .000 – .031 |  | |  |  |  |  |
| Metric invariance | 78.16 | 68 | .991 | .983 | .019 | .000 – .036 | 17.21 | | 8 | .028 |  |  |
| Scalar invariance | 99.78 | 76 | .979 | .964 | .028 | .008 – .042 | 21.99 | | 8 | .005 |  |  |
| Anxiety | | | | | | | | | | | |  |
| Configural invariance | 55.99 | 60 | 1.000 | 1.000 | .000 | .000 – .027 |  | |  |  |  |  |
| Metric invariance | 72.18 | 68 | .997 | .994 | .012 | .000 – .032 | 16.35 | | 8 | .038 |  |  |
| Scalar invariance | 101.36 | 76 | .983 | .970 | .029 | .010 – .043 | 30.48 | | 8 | <.001 |  |  |
| Aesthetics | | | | | | | | | | | |  |
| Configural invariance | 97.23 | 60 | .981 | .958 | .039 | .024 – .053 |  | |  |  |  |  |
| Metric invariance | 105.07 | 68 | .981 | .963 | .037 | .022 – .050 | 8.50 | | 8 | .386 |  |  |
| Scalar invariance | 112.230 | 76 | .981 | .967 | .034 | .020 – .047 | 7.27 | | 8 | .508 |  |  |
| Ideas | | | | | | | | | | | |  |
| Configural invariance | 70.96 | 60 | .991 | .981 | .021 | .000 – .039 |  | |  |  |  |  |
| Metric invariance | 87.14 | 68 | .985 | .970 | .026 | .000 – .042 | 16.62 | | 8 | .034 |  |  |
| Scalar invariance | 102.38 | 76 | .979 | .963 | .029 | .011 – .043 | 14.85 | | 8 | .062 |  |  |

*Note*. CFI = Comparative Fit Index; TLI = Tucker-Lewis Index; RMSEA = Root Mean Square Error of Approximation; CI = Confidence Interval. Satorra-Bentler scaled χ^2^-test were conducted when comparing models. Measurement invariance for the facets Activity, Order, and Depression could not be evaluated because they consisted of only two items.

**Descriptive Results**

Means and standard deviations for all Big Five personality traits for ages 10, 12, 14, and 16 for both sexes are presented in Table S6. As can be seen, mean scores for conscientiousness were significantly higher for girls than boys at all ages, with rather stable mean values at ages 10 and 12 and a slight decrease thereafter. A similar pattern was observed for agreeableness, with significantly higher mean values for girls of all ages. Moreover, agreeableness scores decreased continuously from age 10 to 16 for both sexes. In contrast, neuroticism scores developed differently for girls and boys, with increasing scores for girls from ages 12 to 16, whereas boys’ scores remained stable or even decreased somewhat with increasing age. As a result, sex differences increased from a non-significant Cohen’s *d* of 0.16 at age 10 to 0.65 at age 16, which is considered a medium to large effect size. Extraversion scores were somewhat higher for girls at most time points and showed a decrease from age 14 to 16 for both sexes. Finally, girls showed higher scores on openness at ages 10 and 12 and decreasing trends from age 10 to 16, whereas decreases were less evident among boys, and no sex differences were visible at ages 14 and 16.

**Table S6**

*Means and Standard Deviations for Variables Under Study*

|  | Girls (*n* = 423) | |  | Boys (*n* = 382) | |  | Sex difference | |  | Total (*N* = 805) | |
| --- | --- | --- | --- | --- | --- | --- | --- | --- | --- | --- | --- |
|  | Mean | *SD* |  | Mean | *SD* |  | Cohen’s *d* | *p* |  | Mean | *SD* |
| Conscientiousness age 10 | 3.71 | 0.56 |  | 3.59 | 0.54 |  | 0.20 | .012 |  | 3.65 | 0.55 |
| Conscientiousness age 12 | 3.75 | 0.57 |  | 3.59 | 0.54 |  | 0.28 | .001 |  | 3.68 | 0.57 |
| Conscientiousness age 14 | 3.65 | 0.64 |  | 3.51 | 0.57 |  | 0.23 | .004 |  | 3.58 | 0.61 |
| Conscientiousness age 16 | 3.60 | 0.64 |  | 3.41 | 0.59 |  | 0.32 | <.001 |  | 3.52 | 0.62 |
| Agreeableness age 10 | 4.23 | 0.44 |  | 4.09 | 0.50 |  | 0.26 | .001 |  | 4.16 | 0.48 |
| Agreeableness age 12 | 4.18 | 0.48 |  | 4.05 | 0.48 |  | 0.28 | .001 |  | 4.12 | 0.48 |
| Agreeableness age 14 | 4.08 | 0.49 |  | 3.93 | 0.47 |  | 0.32 | <.001 |  | 4.01 | 0.49 |
| Agreeableness age 16 | 4.02 | 0.52 |  | 3.84 | 0.51 |  | 0.35 | <.001 |  | 3.94 | 0.52 |
| Neuroticism age 10 | 2.57 | 0.59 |  | 2.48 | 0.55 |  | 0.16 | .070 |  | 2.53 | 0.58 |
| Neuroticism age 12 | 2.50 | 0.65 |  | 2.36 | 0.55 |  | 0.23 | .010 |  | 2.43 | 0.61 |
| Neuroticism age 14 | 2.64 | 0.73 |  | 2.30 | 0.61 |  | 0.50 | <.001 |  | 2.48 | 0.69 |
| Neuroticism age 16 | 2.85 | 0.78 |  | 2.36 | 0.69 |  | 0.65 | <.001 |  | 2.63 | 0.78 |
| Extraversion age 10 | 3.61 | 0.53 |  | 3.55 | 0.50 |  | 0.13 | .109 |  | 3.58 | 0.52 |
| Extraversion age 12 | 3.71 | 0.56 |  | 3.56 | 0.54 |  | 0.27 | .002 |  | 3.64 | 0.56 |
| Extraversion age 14 | 3.67 | 0.64 |  | 3.54 | 0.54 |  | 0.20 | .013 |  | 3.61 | 0.60 |
| Extraversion age 16 | 3.44 | 0.72 |  | 3.32 | 0.65 |  | 0.17 | .043 |  | 3.38 | 0.70 |
| Openness age 10 | 3.83 | 0.54 |  | 3.72 | 0.61 |  | 0.20 | .012 |  | 3.78 | 0.58 |
| Openness age 12 | 3.75 | 0.53 |  | 3.57 | 0.61 |  | 0.32 | <.001 |  | 3.66 | 0.58 |
| Openness age 14 | 3.54 | 0.62 |  | 3.49 | 0.58 |  | 0.08 | .317 |  | 3.52 | 0.60 |
| Openness age 16 | 3.38 | 0.63 |  | 3.47 | 0.58 |  | -0.16 | .085 |  | 3.42 | 0.61 |

*Note*. *SD* = standard deviation.

**Intercorrelations among Study Variables**

***Table S7.*** *Intercorrelations for Personality Traits Used in the Study for Boys (under the Diagonal) and Girls (Over the Diagonal)*

|  | (1) | (2) | (3) | (4) | (5) | (6) | (7) | (8) | (9) | (10) | (11) | (12) | (13) | (14) | (15) | (16) | (17) | (18) | (19) | (20) |
| --- | --- | --- | --- | --- | --- | --- | --- | --- | --- | --- | --- | --- | --- | --- | --- | --- | --- | --- | --- | --- |
| (1) Extraversion age 10 |  | 0.51 | 0.42 | 0.40 | -0.37 | -0.17 | -0.12 | -0.12 | 0.31 | 0.18 | 0.15 | 0.09 | 0.27 | 0.19 | 0.14 | 0.10 | 0.33 | 0.21 | 0.13 | 0.10 |
| (2) Extraversion age 12 | 0.43 |  | 0.63 | 0.47 | -0.29 | -0.46 | -0.21 | -0.08 | 0.22 | 0.42 | 0.25 | 0.10 | 0.20 | 0.39 | 0.23 | 0.18 | 0.11 | 0.34 | 0.22 | 0.06 |
| (3) Extraversion age 14 | 0.36 | 0.51 |  | 0.61 | -0.23 | -0.25 | -0.42 | -0.18 | 0.24 | 0.29 | 0.29 | 0.14 | 0.25 | 0.31 | 0.38 | 0.21 | 0.10 | 0.18 | 0.22 | 0.09 |
| (4) Extraversion age 16 | 0.32 | 0.51 | 0.61 |  | -0.20 | -0.21 | -0.19 | -0.37 | 0.10 | 0.10 | 0.13 | 0.13 | 0.15 | 0.17 | 0.20 | 0.26 | 0.09 | 0.11 | 0.12 | 0.19 |
| (5) Neuroticism age 10 | -0.32 | -0.19 | -0.14 | -0.11 |  | 0.45 | 0.36 | 0.32 | -0.39 | -0.32 | -0.19 | -0.13 | -0.38 | -0.33 | -0.24 | -0.24 | -0.15 | -0.06 | 0.00 | 0.01 |
| (6) Neuroticism age 12 | -0.19 | -0.33 | -0.22 | -0.24 | 0.42 |  | 0.53 | 0.41 | -0.26 | -0.50 | -0.31 | -0.18 | -0.19 | -0.51 | -0.30 | -0.29 | -0.02 | -0.15 | -0.08 | 0.01 |
| (7) Neuroticism age 14 | -0.16 | -0.30 | -0.38 | -0.28 | 0.31 | 0.53 |  | 0.51 | -0.31 | -0.37 | -0.48 | -0.23 | -0.18 | -0.30 | -0.41 | -0.28 | 0.04 | -0.02 | -0.04 | 0.07 |
| (8) Neuroticism age 16 | -0.18 | -0.26 | -0.28 | -0.37 | 0.19 | 0.38 | 0.50 |  | -0.10 | -0.20 | -0.25 | -0.27 | -0.15 | -0.16 | -0.22 | -0.36 | 0.11 | 0.11 | 0.09 | 0.02 |
| (9) Conscientiousness age 10 | 0.31 | 0.17 | 0.27 | 0.24 | -0.42 | -0.20 | -0.23 | -0.20 |  | 0.52 | 0.44 | 0.31 | 0.48 | 0.28 | 0.29 | 0.24 | 0.24 | 0.15 | 0.08 | -0.04 |
| (10) Conscientiousness age 12 | 0.12 | 0.31 | 0.22 | 0.25 | -0.29 | -0.34 | -0.24 | -0.20 | 0.42 |  | 0.64 | 0.45 | 0.32 | 0.50 | 0.39 | 0.31 | 0.02 | 0.22 | 0.10 | -0.08 |
| (11) Conscientiousness age 14 | 0.11 | 0.17 | 0.32 | 0.22 | -0.20 | -0.28 | -0.34 | -0.13 | 0.35 | 0.64 |  | 0.63 | 0.20 | 0.27 | 0.45 | 0.31 | -0.02 | 0.09 | 0.16 | -0.04 |
| (12) Conscientiousness age 16 | 0.13 | 0.15 | 0.17 | 0.26 | -0.20 | -0.26 | -0.28 | -0.33 | 0.25 | 0.53 | 0.62 |  | 0.17 | 0.17 | 0.24 | 0.42 | -0.12 | -0.12 | -0.08 | -0.07 |
| (13) Agreeableness age 10 | 0.34 | 0.17 | 0.19 | 0.17 | -0.37 | -0.16 | -0.10 | 0.00 | 0.54 | 0.22 | 0.20 | 0.16 |  | 0.42 | 0.38 | 0.27 | 0.20 | 0.10 | -0.02 | -0.02 |
| (14) Agreeableness age 12 | 0.18 | 0.41 | 0.22 | 0.22 | -0.25 | -0.42 | -0.26 | -0.16 | 0.20 | 0.48 | 0.32 | 0.23 | 0.30 |  | 0.56 | 0.44 | 0.01 | 0.18 | 0.06 | -0.05 |
| (15) Agreeableness age 14 | 0.11 | 0.24 | 0.31 | 0.19 | -0.08 | -0.27 | -0.36 | -0.22 | 0.25 | 0.33 | 0.42 | 0.25 | 0.32 | 0.56 |  | 0.59 | -0.03 | 0.04 | 0.07 | -0.04 |
| (16) Agreeableness age 16 | -0.01 | 0.17 | 0.14 | 0.29 | -0.10 | -0.19 | -0.26 | -0.34 | 0.19 | 0.23 | 0.24 | 0.29 | 0.25 | 0.40 | 0.55 |  | -0.10 | -0.10 | -0.09 | -0.04 |
| (17) Openness age 10 | 0.41 | 0.24 | 0.11 | 0.11 | -0.15 | -0.15 | -0.08 | 0.00 | 0.28 | 0.07 | -0.01 | 0.02 | 0.38 | 0.08 | 0.08 | 0.09 |  | 0.48 | 0.38 | 0.32 |
| (18) Openness age 12 | 0.28 | 0.41 | 0.21 | 0.13 | -0.16 | -0.25 | -0.17 | -0.12 | 0.13 | 0.21 | 0.05 | 0.09 | 0.19 | 0.31 | 0.14 | 0.18 | 0.51 |  | 0.60 | 0.40 |
| (19) Openness age 14 | 0.20 | 0.14 | 0.27 | 0.13 | -0.14 | -0.15 | -0.04 | -0.03 | 0.12 | 0.10 | 0.14 | 0.03 | 0.17 | 0.13 | 0.11 | 0.09 | 0.40 | 0.56 |  | 0.61 |
| (20) Openness age 16 | 0.16 | 0.10 | 0.17 | 0.14 | -0.13 | -0.19 | -0.06 | 0.00 | 0.15 | 0.04 | 0.06 | 0.06 | 0.09 | -0.01 | -0.05 | 0.01 | 0.32 | 0.39 | 0.64 |  |

**Detailed Results from Growth Curve Modelling of the Big Five Personality Traits**

***Table S8.*** *Growth Parameters for Unconditioned Growth Curve Models with Linear and Quadratic Slopes for Big Five Personality Traits*

|  | *Mean* | 95% CI of *Mean* | *p* | *Variance* | 95% CI of *Variance* | *p* |
| --- | --- | --- | --- | --- | --- | --- |
| Intercept | | | | | | |
| Conscientiousness | 3.66 | [3.62; 3.71] | <.001 | 0.14 | [0.06; 0.22] | <.001 |
| Agreeableness | 4.17 | [4.13; 4.21] | <.001 | 0.07 | [0.01; 0.13] | .020 |
| Neuroticism | 2.53 | [2.48; 2.57] | <.001 | 0.18 | [0.08; 0.28] | <.001 |
| Extraversion | 3.58 | [3.54; 3.62] | <.001 | 0.18 | [0.10; 0.25] | <.001 |
| Openness | 3.78 | [3.74; 3.83] | <.001 | 0.21 | [0.14; 0.29] | <.001 |
| Linear slope | | | | | | |
| Conscientiousness | 0.10 | [-0.17; 0.37] | .459 | 2.40 | [-0.22; 5.02] | .072 |
| Agreeableness | -0.32 | [-0.56; -0.07] | .011 | 1.21 | [-0.81; 3.24] | .240 |
| Neuroticism | -0.75 | [-1.08; -0.43] | <.001 | 3.69 | [0.07; 7.31] | .046 |
| Extraversion | 0.74 | [0.47; 1.01] | <.001 | 3.36 | [0.97; 5.75] | .006 |
| Openness | -0.68 | [-0.96; -0.39] | <.001 | 4.26 | [1.81; 6.71] | .001 |
| Quadratic slope | | | | | | |
| Conscientiousness | -0.59 | [-1.01; -0.17] | .006 | 5.90 | [0.61; 11.2] | .029 |
| Agreeableness | -0.12 | [-0.51; 0.26] | .532 | 3.50 | [-1.03; 8.03] | .130 |
| Neuroticism | 1.56 | [1.03; 2.09] | <.001 | 9.18 | [0.96; 17.39] | .029 |
| Extraversion | -1.76 | [-2.20; -1.31] | <.001 | 6.55 | [1.01; 12.10] | .021 |
| Openness | 0.11 | [-0.33; 0.56] | .616 | 12.78 | [7.24; 18.32] | <.001 |

Note. In the growth curves presented here, age is coded such that linear and quadratic slope parameters represent changes during an interval of 10 years. Coding of age was performed in this manner to avoid numerically small estimates. The slope and quadratic growth estimates have to be divided by 10 and 100, respectively, to provide estimates of changes from one year to the next. Estimates of intercepts are not affected by the scaling of age.

**p* < .05. ***p* < .01. ****p* < .001.

***Table S9.*** *Results of Tests for Sex Differences in the Development of Big Five Personality Traits by Using Latent Growth Curves with Sex as Predictor Growth Parameters*

|  | Sex predicting the growth curve intercept  (boys = 0, girls = 1) | | |  | Sex predicting the growth curve linear slope  (boys = 0, girls = 1) | | |  | Sex predicting the growth curve quadratic slope  (boys = 0, girls = 1) | | |
| --- | --- | --- | --- | --- | --- | --- | --- | --- | --- | --- | --- |
|  | *Coef.* | 95% *CI* | *p* |  | *Coef.* | 95% *CI* | *p* |  | *Coef.* | 95% *CI* | *p* |
| Conscientiousness | 0.122 | [0.035; 0.208] | .008 |  | 0.109 | [-0.440; 0.658] | .698 |  | 0.000 | [-0.843; 0.844] | .999 |
| Agreeableness | 0.132 | [0.058; 0.207] | <.001 |  | -0.026 | [-0.524; 0.471] | .917 |  | 0.174 | [-0.605; 0.952] | .662 |
| Neuroticism | 0.078 | [-0.013; 0.170] | .094 |  | 0.246 | [-0.387; 0.880] | .446 |  | 0.759 | [-0.275; 1.794] | .150 |
| Extraversion | 0.071 | [-0.010; 0.152] | .088 |  | 0.390 | [-0.146; 0.925] | .154 |  | -0.550 | [-1.417; 0.318] | .214 |
| Openness | 0.126 | [0.034; 0.218] | .007 |  | 0.353 | [-0.212; 0.918] | .221 |  | -1.185 | [-2.073; -0.297] | .009 |

**References**

Borghuis, J., Denissen, J. J. A., Oberski, D., Sijtsma, K., Meeus, W. H. J., Branje, S., Koot, H. M., & Bleidorn, W. (2017). Big Five Personality Stability, Change, and Codevelopment Across Adolescence and Early Adulthood. *Journal of Personality and Social Psychology*, *113*(4), 641–657. <https://doi.org/10.1037/pspp0000138>

Cheung, G. W., & Rensvold, R. B. (2002). Evaluating Goodness-of-Fit Indexes for Testing Measurement Invariance. *Structural Equation Modeling: A Multidisciplinary Journal*, *9*(2), 233–255. <https://doi.org/10.1207/S15328007SEM0902_5>

de Moor, E. L., Nelemans, S. A., Becht, A. I., Meeus, W., & Branje, S. (2023). Personality Development Across Adolescence and Young Adulthood: The Role of Life Transitions and Self-Concept Clarity. *European Journal of Personality*, *37*(5), 587–604. <https://doi.org/10.1177/08902070221119782>

Little, T. D. (2013). *Longitudinal structural equation modeling*. Guilford.

Little, T. D., Cunningham, W. A., Shahar, G., & Widaman, K. F. (2002). To parcel or not to parcel: Exploring the question, weighing the merits. *Structural Equation Modeling*, *9*(2), 151–173. <https://doi.org/10.1207/S15328007SEM0902_1>

Little, T. D., Rhemtulla, M., Gibson, K., & Schoemann, A. M. (2013). Why the items versus parcels controversy needn’t be one. *Psychological Methods*, *18*(3), 285–300. <https://doi.org/10.1037/a0033266>

Morken, I. S., Wichstrom, L., Steinsbekk, S., & Viddal, K. R. (2024). Depression and Personality Traits Across Adolescence-Within-Person Analyses of a Birth Cohort. *Res Child Adolesc Psychopathol*. <https://doi.org/10.1007/s10802-024-01188-8>

Soto, C. J., John, O. P., Gosling, S. D., & Potter, J. (2008). The developmental psychometrics of big five self-reports: Acquiescence, factor structure, coherence, and differentiation from ages 10 to 20. *Journal of Personality and Social Psychology*, *94*(4), 718–737. <https://doi.org/10.1037/0022-3514.94.4.718>

Widaman, K. F., Ferrer, E., & Conger, R. D. (2010). Factorial invariance within longitudinal structural equation models: Measuring the same construct across time. *Child Development Perspectives*, *4*(1), 10–18. <https://doi.org/10.1111/j.1750-8606.2009.00110.x>

Willroth, E. C., & Atherton, O. E. (2024). Best laid plans: A guide to reporting preregistration deviations. *Advances in Methods and Practices in Psychological Science*, *7*(1). <https://doi.org/10.1177/25152459231213802>
